# Structure of (*E*)-4-amino-5-{[(1,5-dimethyl-3-oxo-2-phenyl-2,3-di­hydro-1*H*-pyrazol-4-yl)imino]­meth­yl}-1-methyl-2-phenyl-2,3-di­hydro-1*H*-pyrazol-3-one: aerial oxidation of 4-amino­anti­pyrine in di­methyl­formamide

**DOI:** 10.1107/S2056989025003676

**Published:** 2025-04-29

**Authors:** R. Kumaravel, Helen Stoeckli-Evans, A. Subashini, M. G. Shankar, Monika Kučeráková, Michal Dušek, Aurélien Crochet, K. Ramamurthi

**Affiliations:** a Department of Physics, Annapoorana Engineering College (Autonomous), Salem - 636308, Tamilnadu, India; b Institute of Physics, University of Neuchâtel, Rue Emile-Argand 11, CH-2000 Neuchâtel, Switzerland; c PG and Research Department of Physics Srimad Andavan Arts and Science College (Autonomous) Affiliated to Bharathidasan University, Tiruchirappalli - 620005 Tamilnadu India; d Department of Physics Swami Dayananda College of Arts and Science Affiliated to Bharathidasan University, Manjakudi - 612610 Tamilnadu India; e Institute of Physics ASCR Na Slovance 2 182 21 Praha 8 Czech Republic; f Chemistry Department, University of Fribourg, Chemin du Musée 9, CH-1700 Fribourg, Switzerland; g Crystal Growth and Thin Film Laboratory Department of Physics Bharathidasan University, Tiruchirappalli - 620024 Tamilnadu India; University of Aberdeen, United Kingdom

**Keywords:** crystal structure, 4-amino­anti­pyrine, aerial oxidation, di­methyl­formamide, hydrogen bonding, Hirshfeld surface

## Abstract

The title compound, C_22_H_22_N_6_O_2_ (**I**), is the result of the aerial oxidation of the 5-methyl group of 4-amino­anti­pyrine to an aldehyde group followed by Schiff base formation with a second mol­ecule of 4-amino­anti­pyrine. The reaction only takes place in the presence of di­methyl­formamide. The central unit of the mol­ecule is close to planar, the pyrazole rings being inclined to each other by 3.74 (15)°. There is an intra­molecular N—H⋯N hydrogen bond enclosing an *S*(6) ring motif and there are two further *S*(6) rings involving weak C—H⋯O=C hydrogen bonds. The mol­ecule has an *E* configuration about the azomethine (—N=CH—) bond. In the crystal, inversion-related mol­ecules are linked by pairs of N—H⋯O hydrogen bonds, forming dimers enclosing *R*^2^_2_(10) loops. The dimers are linked by C—H⋯O hydrogen bonds and C—H⋯π inter­actions, leading to the formation of a three-dimensional supra­molecular network.

## Abstract 2

The formation of the title compound is best explained by the aerial oxidation of the 5-methyl group of 4-amino­anti­pyrine to an aldehyde group, and subsequent inter­molecular Schiff base formation with a second mol­ecule of 4-amino­anti­pyrine. The reaction only takes place in the presence of di­methyl­formamide.

## 1. Chemical context

The crystal structure of 4-amino­anti­pyrine (4-amino-1,5-dimethyl-2-phenyl-1,2-di­hydro-3*H*-pyrazol-3-one, C_11_H_13_N_3_O or ampyrone or 4-AAP) has been reported by Li *et al.* (2013 ▸) and by Mnguni & Lemmerer (2015 ▸), and a co-crystal of 4-AAP has been reported on by Smith & Lemmerer (2019 ▸). The crystal structure of 4-(*N,N*-dimeth­yl)-amino­anti­pyrine was described by Singh & Vijayan (1976 ▸). Derivatives of 4-AAP account for at least two pharmaceutical drugs, amino­anti­pyrine and 4-(*N,N*-dimeth­yl)-amino­anti­pyrine, both of which have been used as analgesics for over a century.

The formation of the title compound, (**I**), is best explained by the aerial oxidation of the 5-methyl group of 4-AAP to an aldehyde group, forming 4-amino-2-methyl-5-oxo-1-phenyl-2,5-di­hydro-1*H*-pyrazole-3-carbaldehyde and subsequent Schiff base formation with a second mol­ecule of 4-amino­anti­pyrine. The reactivity of the methyl group is indicated by the hyperconjugative effect and oxidation to the aldehyde by air takes place in the presence of di­methyl­formamide (DMF). The role of DMF in chemistry has been reviewed by Heravi *et al.* (2018 ▸). They noted that DMF has been used as a reagent in a number of important organic reactions, such as the Vilsmeier–Haack reaction (Vilsmeier & Haack, 1927 ▸), which result in the formyl­ation of hetero-aromatic compounds
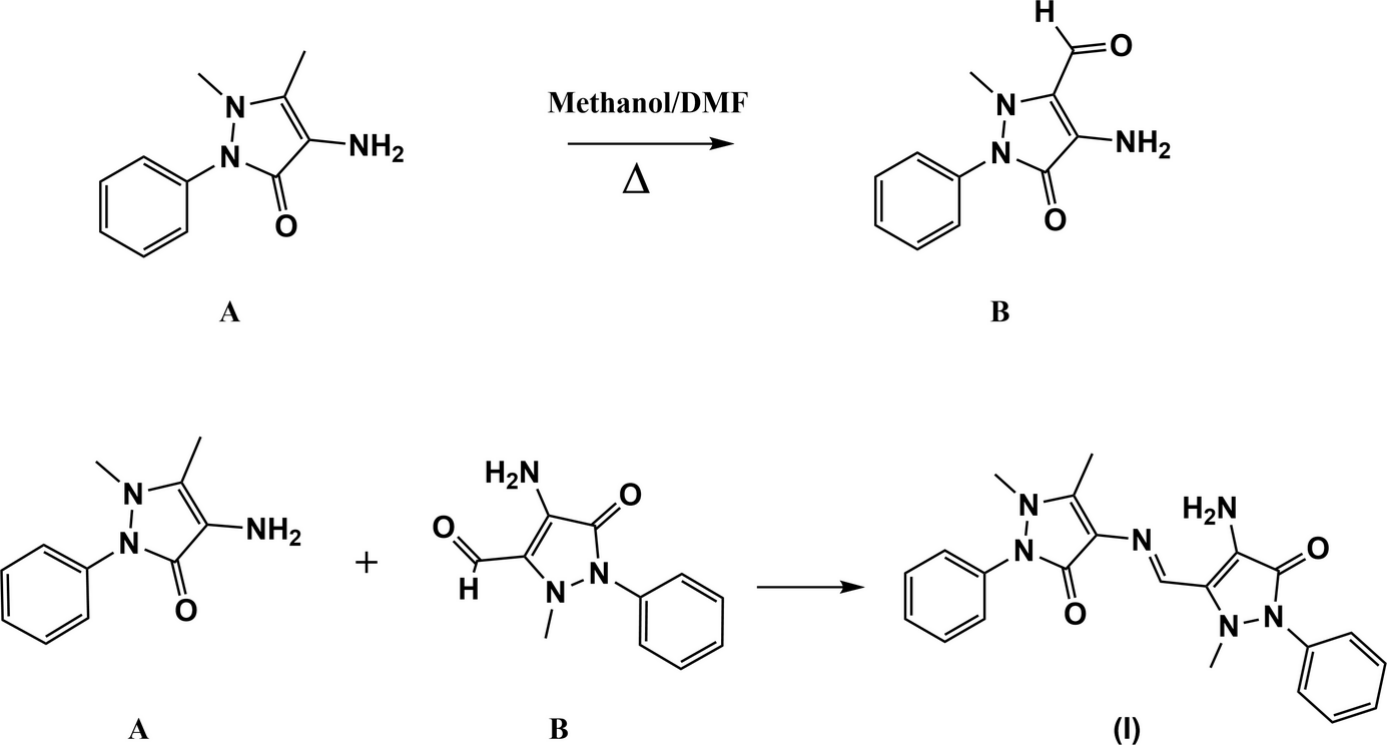
.

Compound (**I**) was produced serendipitously when attempts were made to form CdCl_2_ or HgCl_2_ complexes of 4-amino­anti­pyrine (Stoeckli-Evans *et al.*, 2025 ▸), when di­methyl­formamide (DMF) was added to the reaction mixture. When the reaction was repeated in various solvents in the absence of the metal halide, for example with methanol and aceto­nitrile or methanol and acetone, no reaction took place. It was found that compound (**I**) was only formed when DMF was present as one of the solvents.

To the best of our knowledge, the aerial oxidation of an amino­anti­pyrine was first reported by Kametani *et al.* (1967 ▸). A yellow substance was formed as a byproduct when studying the fusion of 4-(*N,N*-dimeth­yl)-amino­anti­pyrine with barbital in the presence of air. To obtain a large amount of this yellow compound the reaction was repeated by introducing air into a heated solution of the amino­anti­pyrine in different solvents, such as ethanol, acetic acid or acetic anhydride. They showed by NMR and IR spectroscopic analyses that they had produced the aldehyde, 4-(di­methyl­amino)-2-methyl-5-oxo-1-phenyl-2,5-di­hydro-1*H*-pyrazole-3-carbaldehyde (Kametani *et al.*, 1967 ▸).

The result of aerial oxidation of the 5-methyl group of certain 4-amino­anti­pyrine Schiff bases to form *in situ* —CH_2_OH, —CH(OH)_2_ and —COOH groups has been observed in the formation of various copper(II) and cobalt(II) complexes (see §4. *Database survey*).

## 2. Structural commentary

**Figure 1:**
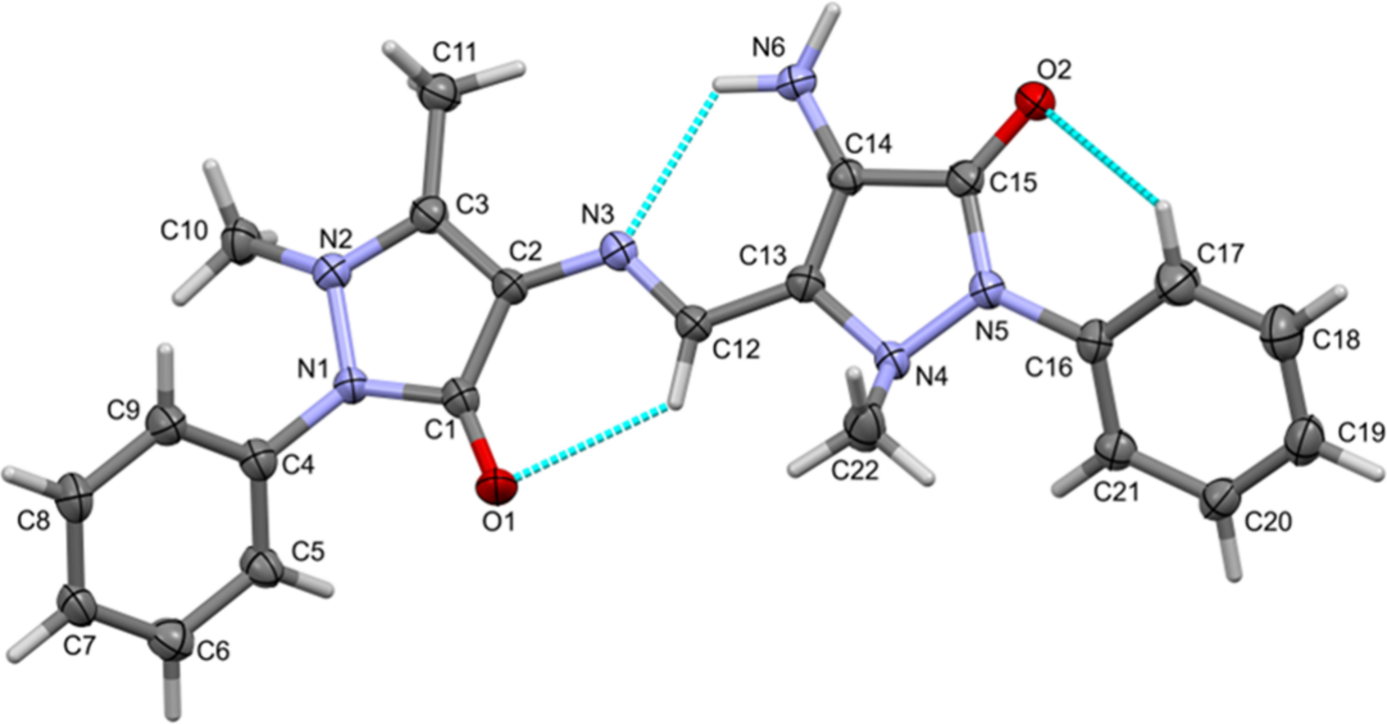
A view of the mol­ecular structure of (**I**) with atom labelling and displacement ellipsoids drawn at the 50% probability level.

The mol­ecular structure of (**I**) is illustrated in Fig. 1 ▸. The central unit is close to planar with the pyrazole rings, N1/N2/C1–C3 (r.m.s. deviation = 0.036 Å) and N4/N5/C13–C15 (r.m.s. deviation = 0.035 Å), being inclined to each other by 3.74 (15)°. The planarity of the central unit is consolidated by the presence of an intra­molecular N6—H6*AN*⋯N3 hydrogen bond (Table 1 ▸), which generates an *S*(6) ring motif, as do two C—H⋯O=C hydrogen bonds (Table 1 ▸ and Fig. 1 ▸).

The title mol­ecule has an *E* configuration about the azomethine (—N3=C12H—) bond whose bond length is 1.297 (3) Å. This is slightly longer than the average value of 1.286 (6) Å (see §4. *Database survey*). The arene ring C4–C9 is inclined to the pyrazole ring N1/N2/C1–C3 mean plane by 55.63 (14)°, while the arene ring C16–C21 is inclined to the pyrazole ring N4/N5/C13–C15 mean plane by 32.84 (15)°. The arene rings are inclined to each other by 44.19 (4)°.

The sum of the angles subtended by the methyl substituted atom N2 is 354.9°. In contrast, the methyl-substituted N atom, N4, has a definite pyramidal geometry with the corresponding sum of angles being 328.8°. This later value is more typical and is similar to the value of 332.4° reported for 4-AAP (CSD refcode LOYXEE; Mnguni & Lemmerer, 2015 ▸). See also §4. *Database survey*.

## 3. Supra­molecular features

**Figure 2:**
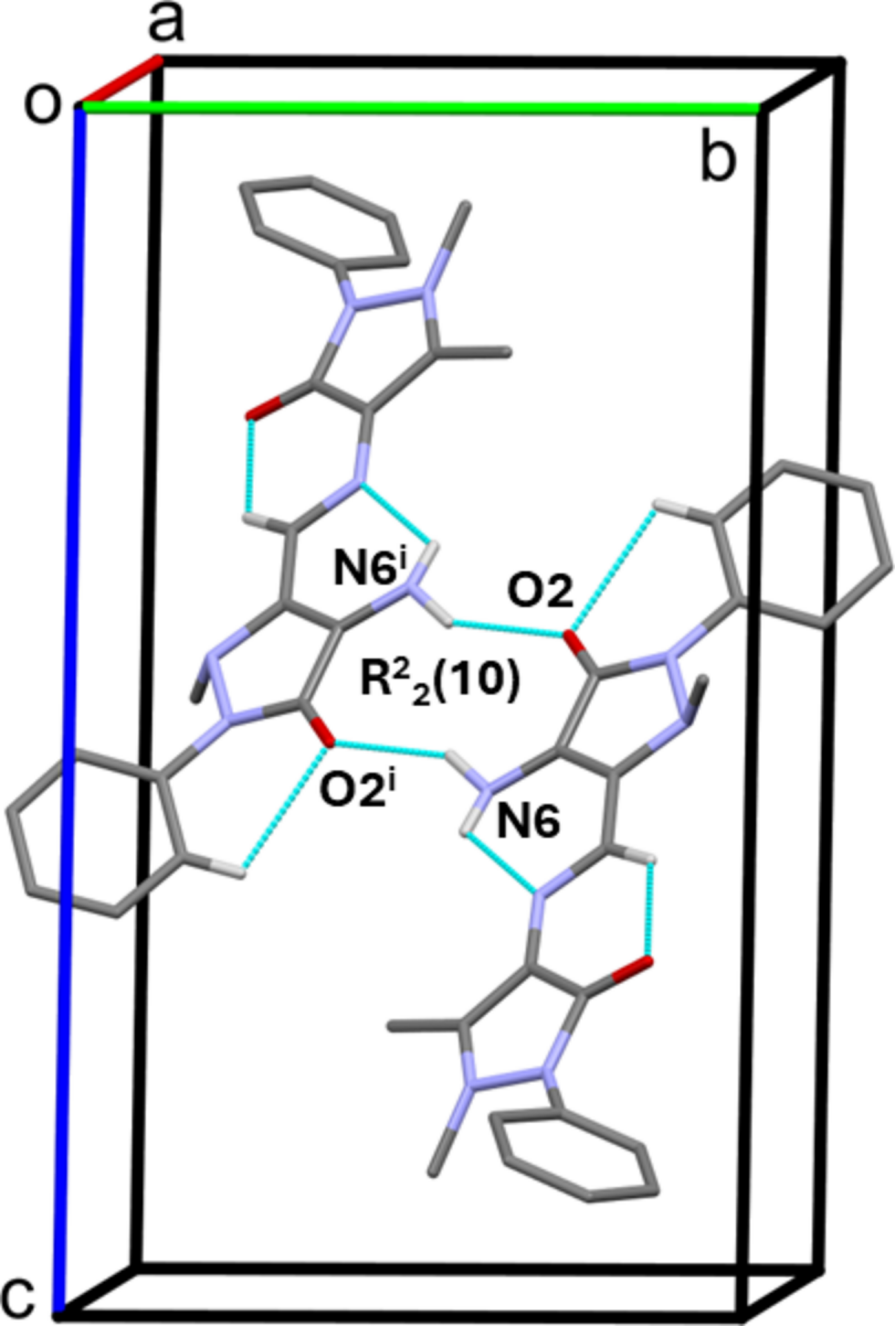
A partial view along the *a* axis of the crystal packing of (**I**). Inversion-related mol­ecules are linked by a pair of N—H⋯O hydrogen bonds (Table 1 ▸), forming a dimer enclosing an 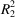
(10) ring motif.

**Figure 3:**
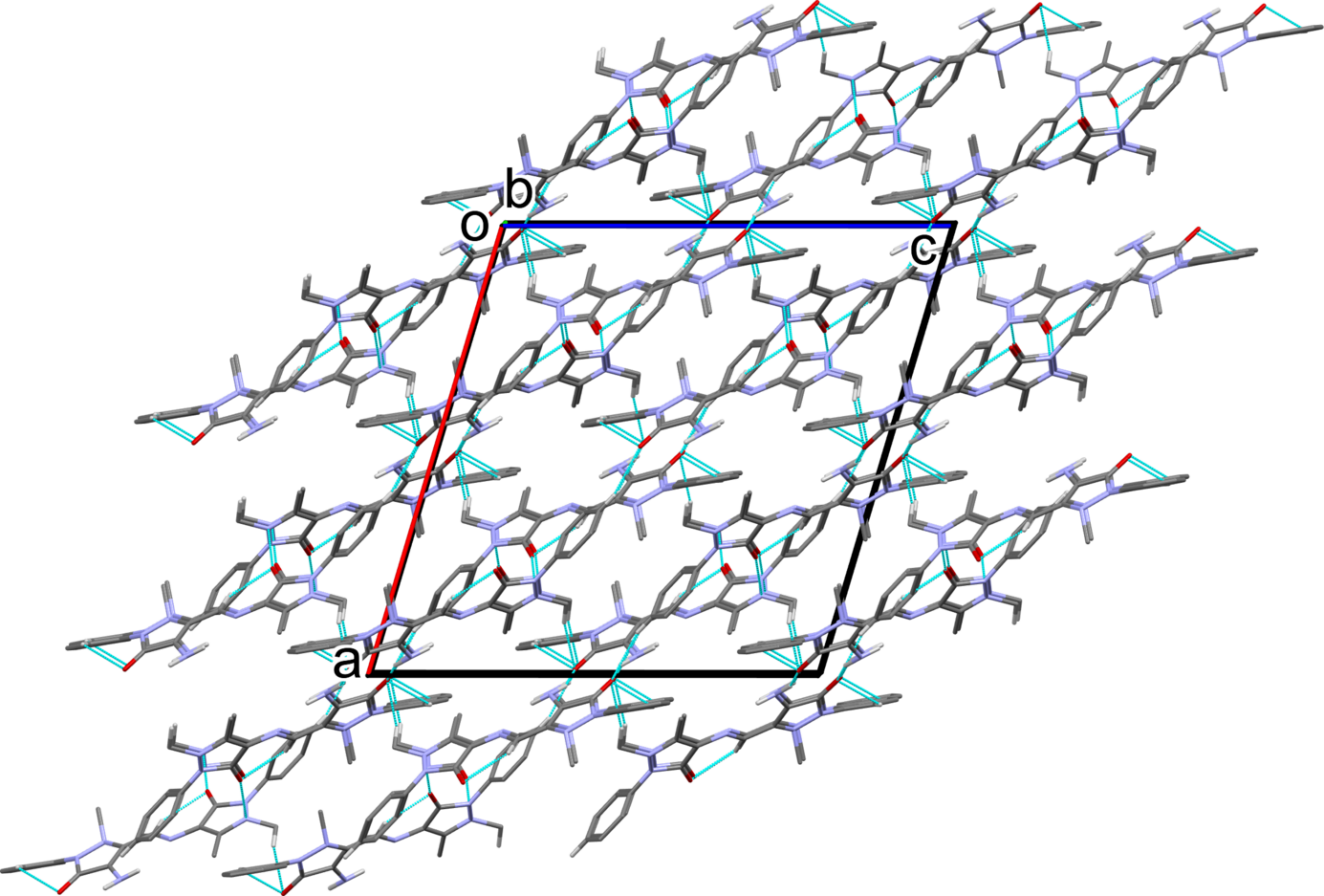
A view along the *b* axis of the crystal packing of (**I**). For clarity, only the H atoms involved in hydrogen bonding (Table 1 ▸) have been included.

In the crystal of (**I**), inversion-related mol­ecules are linked by pairwise N—H⋯O hydrogen bonds, forming dimers enclosing an 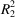
(10) ring motif (Table 1 ▸, Fig. 2 ▸). The dimers are linked by C—H⋯O hydrogen bonds and C—H⋯π inter­actions (Table 1 ▸), forming a three-dimensional supra­molecular network (Fig. 3 ▸).

## 4. Database survey

A search of the Cambridge Structural Database (CSD, V5.46 update February 2025; Groom *et al.*, 2016 ▸) for the 4-AAP moiety gave 582 hits; 413 are organic compounds and 169 are metal–organic complexes. In a series of copper(II) and cobalt(II) complexes, *in situ* oxidation of the 5-methyl group of the pyrazole moiety takes place. For the copper(II) complexes (CSD refcodes: CIHPUF; Wang & Zheng, 2007 ▸ and JAXSOU; Parvarinezhad *et al.*, 2022 ▸) the transformation is to an alcohol (—CH_2_OH). For the cobalt(II) complexes three transformations have been observed, to —CH_2_OH, —CH(OH)_2_ and —COOH (CSD refcodes: JUNMAI, JUNMEM and JUNMIQ; Loukopoulos *et al.*, 2015 ▸). Full details and references of the CSD search are given in the supporting information.

A search of the CSD for Schiff base derivatives of 4-AAP with an —N=C— bond (with the following restrictions: three-dimensional coordinates determined, *R* factor ≤ 0.075, no disorder, no errors, not polymeric, no ions, single crystals only and only organics) yielded 208 hits. The analysis in *Mercury* (Macrae *et al.*, 2020 ▸) of the —N=C— bond length found that it varies from 1.256 to 1.300 Å, with a mean value of 1.282 (6) Å. In (**I)** the N3=C12 bond length is slightly longer at 1.297 (3) Å. The dihedral angles involving the phenyl ring and the mean plane of the pyrazole ring vary from 32.4 to 80.2°, with an average value of 52.32 (15)°. As noted above, the corresponding values observed for the two 4-AAP moieties in (**I**) are 55.63 (14) and 32.84 (15)°, the latter dihedral angle being close to the lower limit value.

The pyramidal geometry of the methyl-substituted N atom of the pyrazole ring, measured by the sum of the three angles involving the N atom, was found to have a lower limit of *ca* 340° in the two independent mol­ecules of 4-((*E*)-{2-[*N*-(1,5-dimethyl-3-oxo-2-phenyl-2,3-di­hydro-1*H*-pyrazol-4-yl)-carb­oximido­yl]benzyl­idene}amino)-1,5-dimethyl-2-phenyl-2,3-di­hydro-1*H*-pyrazol-3-one (ABADEO; Potgieter *et al.*, 2011 ▸), and a higher limit of 359.0° in {2-[(1,5-dimethyl-2-phenyl-3-oxo-2,3-di­hydro-1*H*-pyrazol-4-yl­imino)­meth­yl]-phen­oxy}acetic acid methanol hemisolvate (EVILOK; You *et al.*, 2004 ▸). In (**I**), the sum of these angles is *ca* 328.8° for atom N4, hence this atom is highly pyramidal (Fig. 1 ▸), more so than for the N atoms in ABADEO. The value of *ca* 354.9° for atom N2 is close to the value of 359.0° observed in EVILOK.

**Figure 4:**
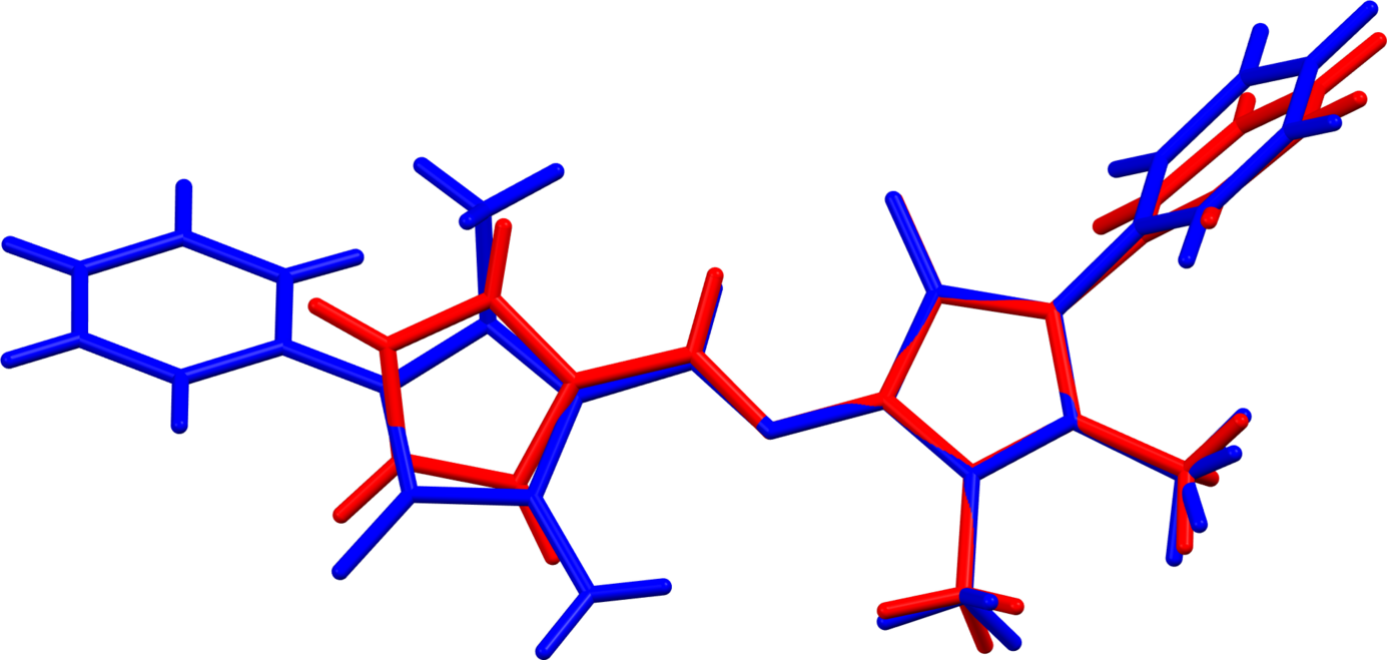
A view of the structural overlay of (**I**) and DEXTAC (Jing & Chen, 2007 ▸); the r.m.s. deviation is 0.018 Å (*Mercury*; Macrae *et al.*, 2020 ▸).

Using the CSD Python API, mol­ecular similarity, only one compound was found when compared to the structure of compound (**I**), namely 1,5-dimethyl-2-phenyl-4-[(1*H*-pyrrol-2-yl-methyl­ene)amino]­pyrazol-3(2*H*)-one (DEXTAC; Jing & Chen, 2007 ▸), which has a similarity index of 0.726. The structural overlap of the two compounds is shown in Fig. 4 ▸; the r.m.s. deviation is 0.018 Å (*Mercury*; Macrae *et al.*, 2020 ▸). Here, the —N=C— bond length is 1.282 (1) Å and the dihedral angle involving the phenyl ring and the mean plane of the pyrazole ring is 62.5 (1)°.

## 5. Hirshfeld surface analysis and two-dimensional fingerprint plots

**Figure 5:**
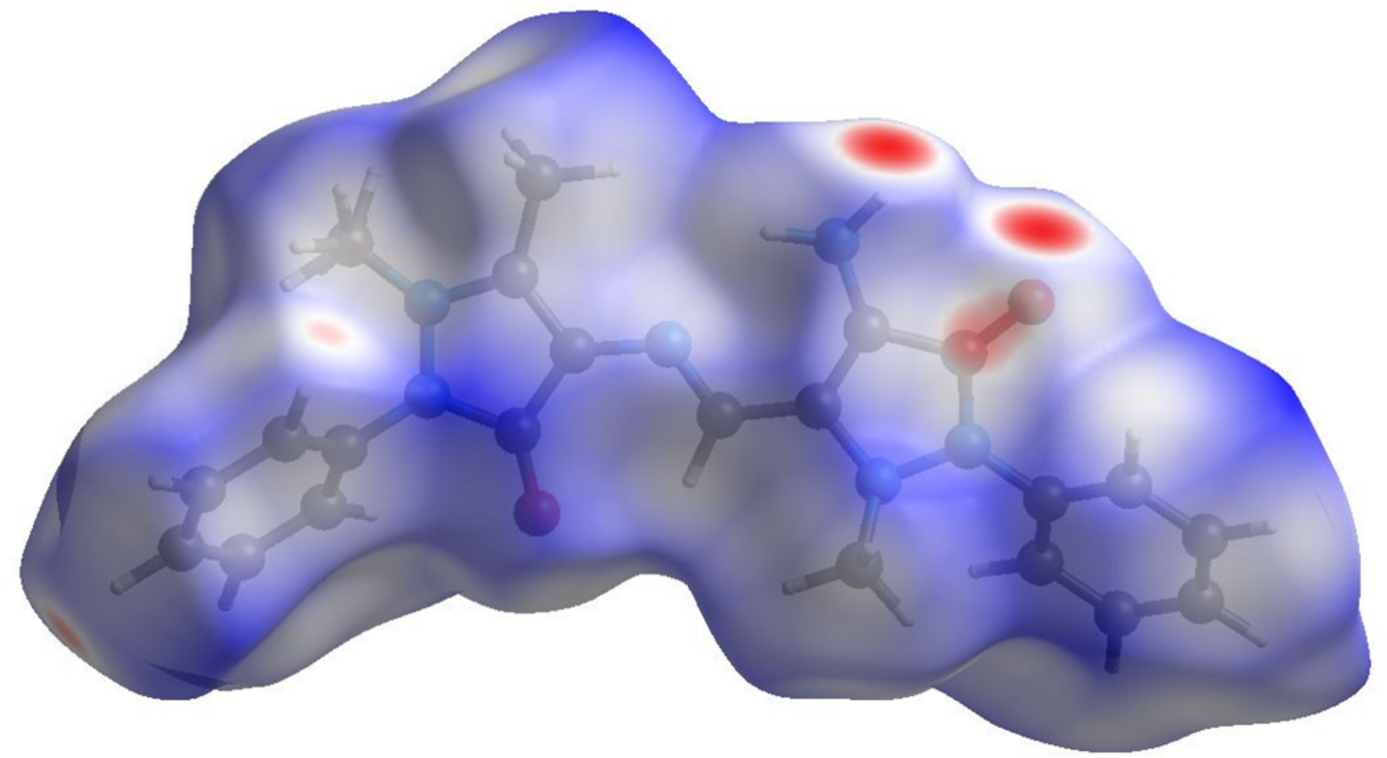
The Hirshfeld surface of (**I**), mapped over *d*_norm_.

The Hirshfeld surface analysis and the associated two-dimensional fingerprint plots were generated with *CrystalExplorer17* (Spackman *et al.*, 2021 ▸) and interpreted following the protocol of Tan *et al.* (2019 ▸). The Hirshfeld surface for compound (**I**) is illustrated in Fig. 5 ▸. The presence of prominent red spots indicates that short contacts are particularly significant in the crystal packing.

**Figure 6:**
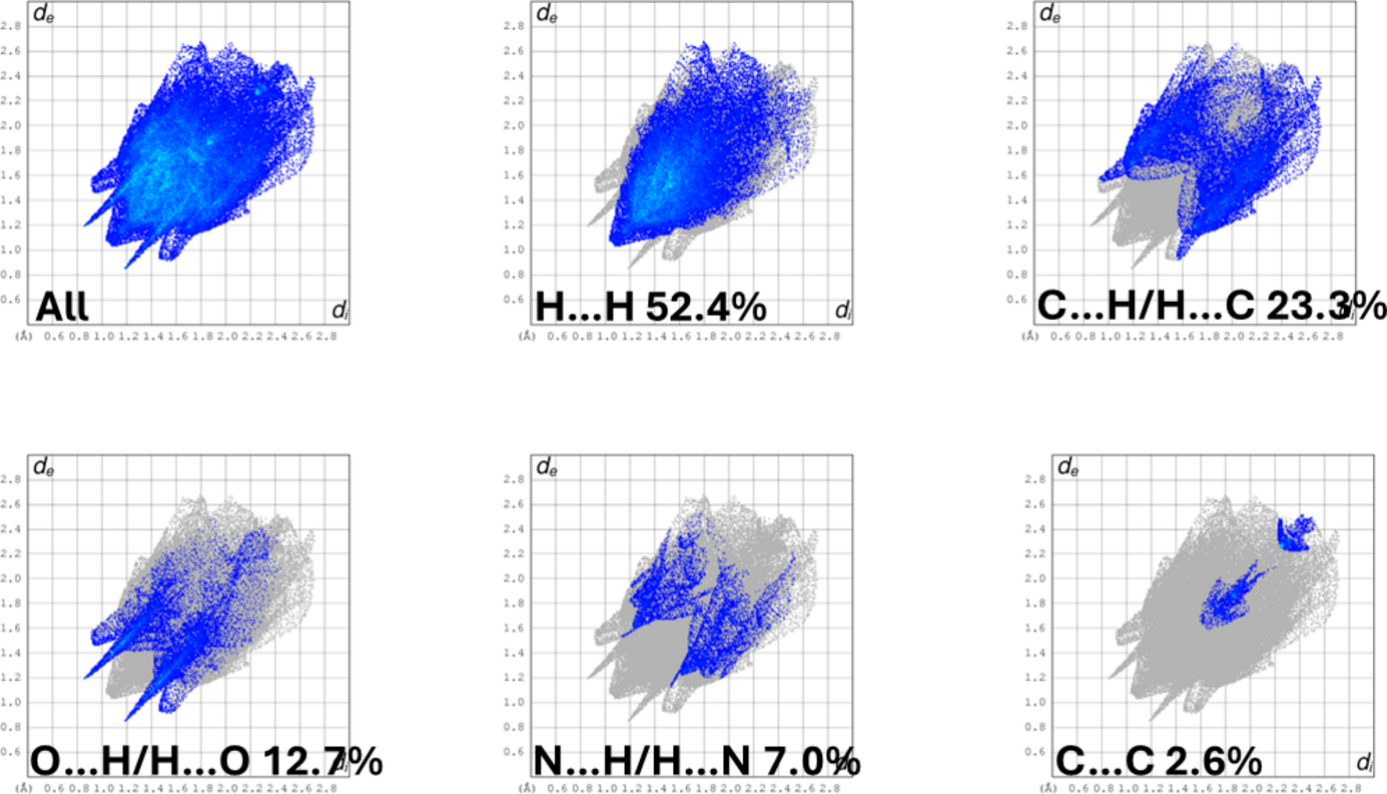
The full two-dimensional fingerprint plot for (**I**), and those delineated into H⋯H, C⋯H/H⋯C, O⋯H/H⋯O, N⋯H/H⋯N and C⋯C contacts.

The full two-dimensional fingerprint plots for (**I**) are given in Fig. 6 ▸. The H⋯H contacts have a major contribution of 52.4% to the Hirshfeld surface. The second most significant contribution is from the C⋯H/H⋯C contacts at 23.3%. The O⋯H/H⋯O contacts contribute 12.7% and have sharp pincer-like spikes at *d_e_ + d_i_* ≃ 2.1 Å. The N⋯H/H⋯N contacts contribute 7.0%, and the C⋯C contacts contribute 2.6%. These values can be correlated with the various hydrogen bonds and other inter­atomic inter­actions in the crystal (Table 1 ▸).

## 6. Energy frameworks

**Figure 7:**
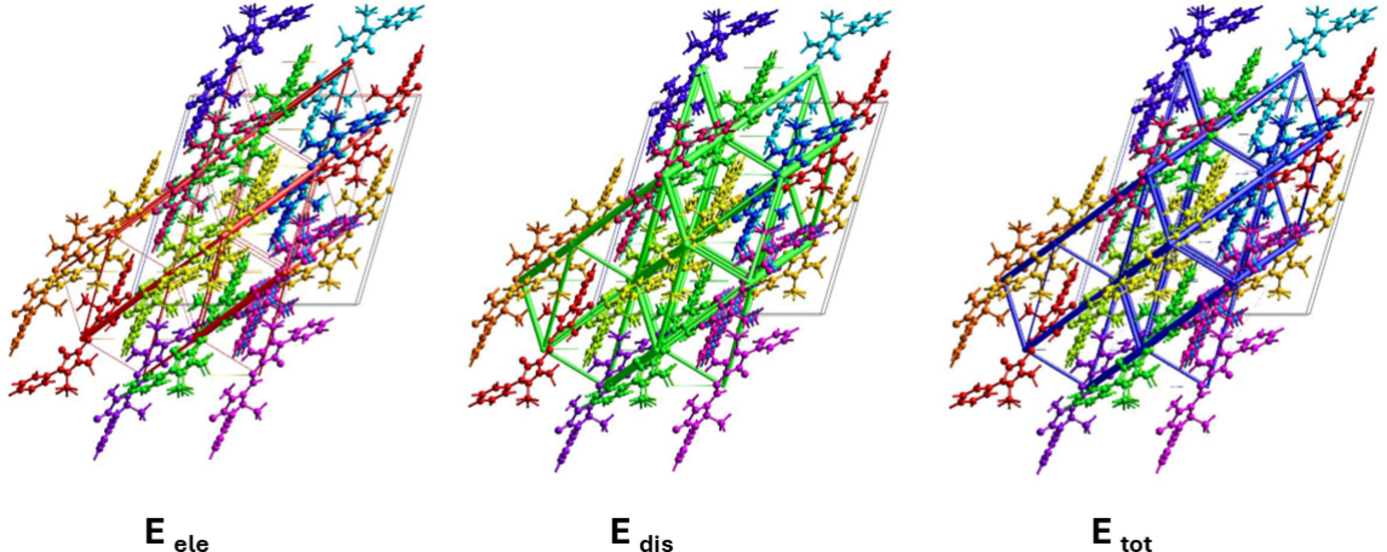
The energy frameworks calculated for (**I**), viewed along the *b*-axis direction, showing the electrostatic potential forces (*E*_ele_), the dispersion forces (*E*_dis_) and the total energy diagram (*E*_tot_).

A comparison of the energy frameworks calculated for (**I**), showing the electrostatic potential forces (*E*_ele_), the dispersion forces (*E*_dis_) and the total energy diagrams (*E*_tot_), are shown in Fig. 7 ▸. The energies were obtained by using wave functions at the HF/3-2IG level of theory. The cylindrical radii are proportional to the relative strength of the corresponding energies (Spackman *et al.*, 2021 ▸; Tan *et al.*, 2019 ▸). They have been adjusted to the same scale factor of 90 with a cut-off value of 6 kJ mol^−1^ within a radius of 3.8 Å of a central reference mol­ecule.

The major contribution to the inter­molecular inter­actions is from dispersion forces (*E*_dis_), as expected in view of the significant contribution to the HS of the H⋯H contacts at 52.4%. The colour-coded inter­action mapping within a radius of 3.8 Å of a central reference mol­ecule and the various contributions to the total energy (*E*_tot_) for compound (**I**) are given in Fig. S1 of the supporting information.

## 7. Synthesis, spectroscopic data and thermal analysis

A solution of 0.100 mmol of 4-amino­anti­pyrine and an equimolar qu­antity of cadmium chloride (or mercury chloride) in a solvent mixture of 5 ml of methanol and 5 ml of dimethyl formamide (DMF) was refluxed at 363 K for 6 h using an oil bath. The solution was then left at room temperature for 10 days. On evaporation of the solvents reddish-brown crystals of (**I**) were obtained; m.p. 517–518 K. The same compound was obtained when 4-amino­anti­pyrine was refluxed in methanol and DMF in the absence of the metal chloride. When 4-amino­anti­pyrine was heated in a mixture of different solvents, it was observed that compound (**I**) was only produced in the presence of DMF.

**Figure 8:**
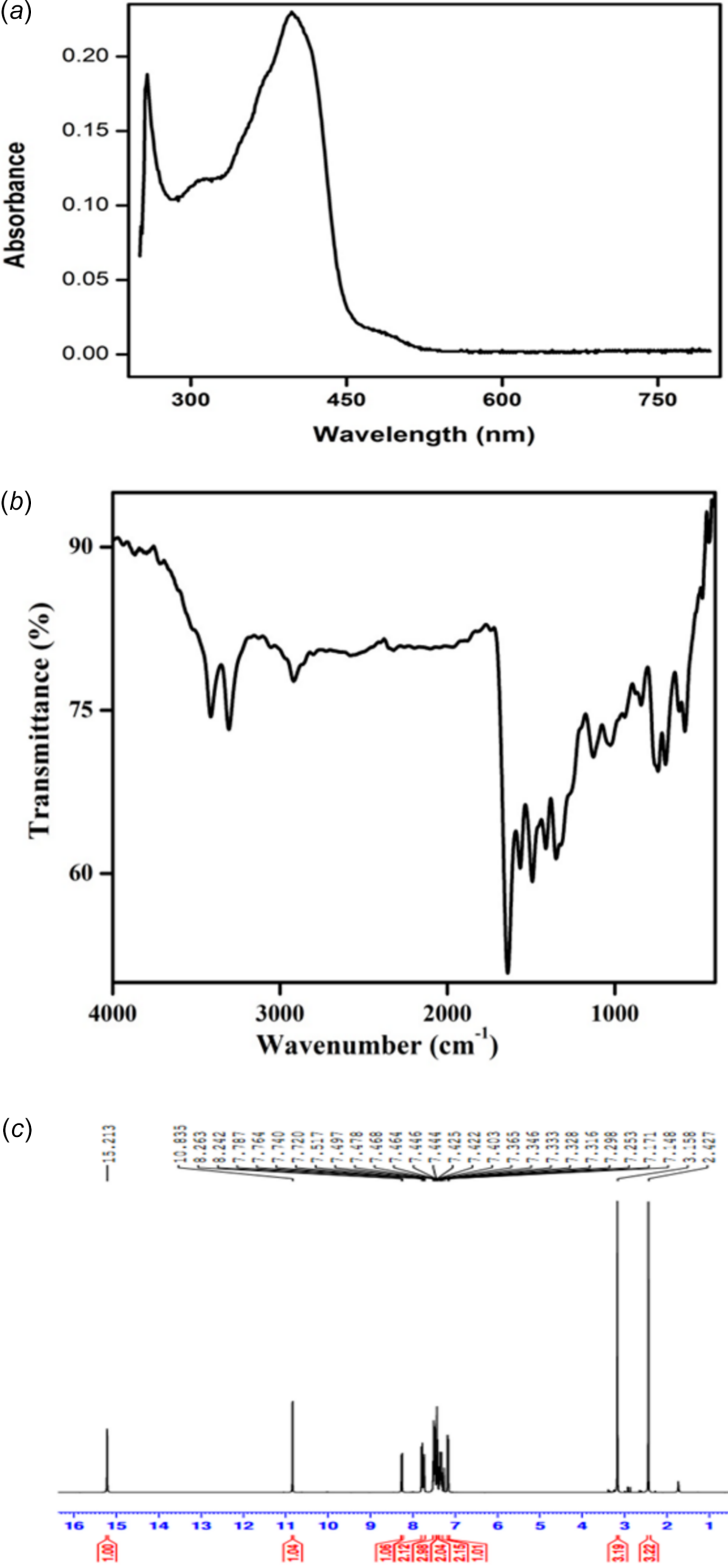
(*a*) The UV-vis spectrum of (**I**) in the range 250–800 nm, (*b*) the FTIR spectrum of (**I**) in the range 400–4000 cm^−1^, and (*c*) the ^1^H NMR spectrum of (**I)**.

The absorption spectrum of (**I**) was measured using a UV-Vis spectrometer in the wavelength range of 200–800 nm in CHCl_3_ as solvent (Fig. 8 ▸*a*). The absorption band at 258 nm is attributed to the π–π^*^ transitions of the aromatic rings. The second absorption band at 397 nm is due to *n*–π^*^ transitions of the C=O and C=N bonds.

The FTIR spectrum of (**I**) was recorded using a JASCO Infrared spectrometer (KBr pellet) between 400–4000 cm^−1^ (Fig. 8 ▸*b*). For the spectrum of 4-amino­anti­pyrine, see Swaminathan *et al.* (2009 ▸). A prominent absorption peak at 1569 cm^−1^ corresponding to the C=N stretching frequency confirms the formation of the Schiff base compound (**I**). For the NH_2_ group of 4-amino­anti­pyrine, strong symmetric and asymmetric stretching vibrations are observed at 3326 and 3432 cm^−1^. In compound (**I**), these vibrations are displaced and appear as medium-sized peaks at 3313 and 3421 cm^−1^. The C=O stretching frequency for 4-amino­anti­pyrine appears at 1679 cm^−1^, while for compound (**I**) this vibrational frequency is red shifted to 1648 cm^−1^. The shifts in the N—H and C=O stretching frequencies suggest a significant conjugation between these functional groups.

The ^1^H NMR spectrum of (**I**) was recorded in CDCl_3_ using a Bruker AC 400 MHz-NMR spectrometer (Fig. 8 ▸*c*). The peaks at 2.43 ppm and 3.15 ppm correspond to the methyl groups (—CH_3_) attached to the carbon and nitro­gen atoms, respectively, of the pyrazole rings. The aromatic protons appear in their usual range of 7.15–7.8 ppm, while the methine H atom (H12) resonates at 8.25 ppm confirming the Schiff base formation. As shown by Hansen & Spanget-Larsen (2017 ▸), the presence of intra­molecular hydrogen bonding has a significant effect on the chemical shifts of the H atoms involved. The presence of the strong intra­molecular N—H⋯N hydrogen bond (N6—H6*AN*⋯N3) results in a shift to 15.22 ppm for this N—H proton. The resonance at 10.83 ppm can be assigned to the second H atom of the NH_2_ group.

An SQT Q600 V20.9 Build 20 Simultaneous Thermo Analytical system was used to measure the TGA/DTA of (**I**) in a nitro­gen atmosphere with a heating rate of 10°C min^−1^ (Fig. S2 of the supporting information). The TGA curve for (**I)** reveals a single-stage weight loss starting around 136°C. The compound decomposes before reaching its melting point as indicated by the DTA curve.

## 8. Refinement

Crystal data, data collection and structure refinement details are summarized in Table 2 ▸. The amino H atoms (NH_2_) were located in a difference-Fourier map and freely refined. The C-bound H atoms were included in calculated positions and refined as riding, with C—H = 0.95–0.98 Å and *U*_iso_(H) = 1.2*U*_eq_(C) or 1.5*U*_eq_(methyl C). The H atoms of methyl group C10 were modelled as disordered over two orientations with an AFIX 123 constraint in *SHELXL*.

## Supplementary Material

Crystal structure: contains datablock(s) I, global. DOI: 10.1107/S2056989025003676/hb8135sup1.cif

Structure factors: contains datablock(s) I. DOI: 10.1107/S2056989025003676/hb8135Isup2.hkl

Supplementary figures and CSD Search. DOI: 10.1107/S2056989025003676/hb8135sup3.pdf

Supporting information file. DOI: 10.1107/S2056989025003676/hb8135Isup4.cml

CCDC reference: 2446090

Additional supporting information:  crystallographic information; 3D view; checkCIF report

## Figures and Tables

**Table 1:** Hydrogen-bond geometry (Å, °) *Cg*1 is the centroid of C4–C9 phenyl ring.

*D*—H⋯*A*	*D*—H	H⋯*A*	*D*⋯*A*	*D*—H⋯*A*
N6—H6*AN*⋯N3	0.90 (4)	2.30 (4)	2.894 (3)	123 (3)
N6—H6*BN*⋯O2^i^	0.98 (3)	2.09 (3)	2.953 (3)	147 (3)
C7—H7⋯O2^ii^	0.95	2.49	3.417 (4)	165
C10—H10*D*⋯O2^iii^	0.98	2.52	3.472 (4)	164
C12—H12⋯O1	0.95	2.34	3.022 (3)	128
C17—H17⋯O2	0.95	2.55	3.013 (4)	110
C11—H11*B*⋯*Cg*1^iii^	0.98	2.96	3.645 (3)	128

Symmetry codes: (i) 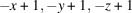; (ii) 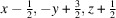; (iii) 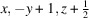.

**Table 2:** Experimental details

Crystal data	
Chemical formula	C_22_H_22_N_6_O_2_
*M*_r_	402.45
Crystal system, space group	Monoclinic, *C*2/*c*
Temperature (K)	95
*a*, *b*, *c* (Å)	20.2393 (11), 10.6519 (8), 19.4225 (11)
β (°)	106.614 (5)
*V* (Å^3^)	4012.4 (4)
*Z*	8
Radiation type	Cu *K*α
μ (mm^−1^)	0.73
Crystal size (mm)	0.11 × 0.06 × 0.02

Data collection	
Diffractometer	SuperNova, Dual, Cu at home/near, AtlasS2
Absorption correction	Multi-scan (*CrysAlis PRO*; Rigaku OD, 2020 ▸)
*T*_min_, *T*_max_	0.489, 1.000
No. of measured, independent and observed [*I* > 2σ(*I*)] reflections	11696, 3915, 2695
*R*_int_	0.078
(sin θ/λ)_max_ (Å^−1^)	0.621

Refinement	
*R*[*F*^2^ > 2σ(*F*^2^)], *wR*(*F*^2^), *S*	0.058, 0.137, 1.09
No. of reflections	3915
No. of parameters	281
No. of restraints	3
H-atom treatment	H atoms treated by a mixture of independent and constrained refinement
Δρ_max_, Δρ_min_ (e Å^−3^)	0.23, −0.24

Computer programs: *CrysAlis PRO* (Rigaku OD, 2020 ▸), *SUPERFLIP* (Palatinus & Chapuis, 2007 ▸; *JANA2020* (Petříček *et al.*, 2023 ▸), *SHELXL2019/3* (Sheldrick, 2015 ▸), *Mercury* (Macrae *et al.*, 2020 ▸),*PLATON* (Spek, 2020 ▸) and *publCIF* (Westrip, 2010 ▸).

## supplementary crystallographic information

### (E)-4-Amino-5-{[(1,5-dimethyl-3-oxo-2-phenyl-2,3-dihydro-1H-pyrazol-4-yl)imino]methyl}-1-methyl-2-phenyl-2,3-dihydro-1H-pyrazol-3-one Crystal data

**Table:**

C_22_H_22_N_6_O_2_	*F*(000) = 1696
*M**_r_* = 402.45	*D*_x_ = 1.332 Mg m^−^^3^
Monoclinic, *C*2/*c*	Cu *K*α radiation, λ = 1.54184 Å
*a* = 20.2393 (11) Å	Cell parameters from 2185 reflections
*b* = 10.6519 (8) Å	θ = 4.5–73.1°
*c* = 19.4225 (11) Å	µ = 0.73 mm^−^^1^
β = 106.614 (5)°	*T* = 95 K
*V* = 4012.4 (4) Å^3^	Block, yellow
*Z* = 8	0.11 × 0.06 × 0.02 mm

### (E)-4-Amino-5-{[(1,5-dimethyl-3-oxo-2-phenyl-2,3-dihydro-1H-pyrazol-4-yl)imino]methyl}-1-methyl-2-phenyl-2,3-dihydro-1H-pyrazol-3-one Data collection

**Table:**

SuperNova, Dual, Cu at home/near, AtlasS2 diffractometer	3915 independent reflections
Radiation source: micro-focus sealed X-ray tube	2695 reflections with *I* > 2σ(*I*)
Mirror monochromator	*R*_int_ = 0.078
Detector resolution: 5.2027 pixels mm^-1^	θ_max_ = 73.4°, θ_min_ = 4.6°
ω scans	*h* = −20→24
Absorption correction: multi-scan (CrysAlisPro; Rigaku OD, 2020)	*k* = −13→11
*T*_min_ = 0.489, *T*_max_ = 1.000	*l* = −21→24
11696 measured reflections	

### (E)-4-Amino-5-{[(1,5-dimethyl-3-oxo-2-phenyl-2,3-dihydro-1H-pyrazol-4-yl)imino]methyl}-1-methyl-2-phenyl-2,3-dihydro-1H-pyrazol-3-one Refinement

**Table:**

Refinement on *F*^2^	Primary atom site location: structure-invariant direct methods
Least-squares matrix: full	Secondary atom site location: difference Fourier map
*R*[*F*^2^ > 2σ(*F*^2^)] = 0.058	Hydrogen site location: mixed
*wR*(*F*^2^) = 0.137	H atoms treated by a mixture of independent and constrained refinement
*S* = 1.09	*w* = 1/[σ^2^(*F*_o_^2^) + (0.0162*P*)^2^ + 0.9186*P*] where *P* = (*F*_o_^2^ + 2*F*_c_^2^)/3
3915 reflections	(Δ/σ)_max_ < 0.001
281 parameters	Δρ_max_ = 0.23 e Å^−^^3^
3 restraints	Δρ_min_ = −0.24 e Å^−^^3^

### (E)-4-Amino-5-{[(1,5-dimethyl-3-oxo-2-phenyl-2,3-dihydro-1H-pyrazol-4-yl)imino]methyl}-1-methyl-2-phenyl-2,3-dihydro-1H-pyrazol-3-one Special details

**Table:**

Geometry. All esds (except the esd in the dihedral angle between two l.s. planes) are estimated using the full covariance matrix. The cell esds are taken into account individually in the estimation of esds in distances, angles and torsion angles; correlations between esds in cell parameters are only used when they are defined by crystal symmetry. An approximate (isotropic) treatment of cell esds is used for estimating esds involving l.s. planes.

### (E)-4-Amino-5-{[(1,5-dimethyl-3-oxo-2-phenyl-2,3-dihydro-1H-pyrazol-4-yl)imino]methyl}-1-methyl-2-phenyl-2,3-dihydro-1H-pyrazol-3-one Fractional atomic coordinates and isotropic or equivalent isotropic displacement parameters (Å^2^)

**Table:**

	*x*	*y*	*z*	*U*_iso_*/*U*_eq_	Occ. (<1)
O1	0.26471 (10)	0.82349 (18)	0.71602 (11)	0.0333 (5)	
O2	0.48785 (10)	0.67524 (17)	0.45511 (10)	0.0270 (4)	
N1	0.28813 (11)	0.6763 (2)	0.80851 (12)	0.0241 (5)	
N2	0.32274 (11)	0.5621 (2)	0.82181 (12)	0.0239 (5)	
N3	0.36940 (11)	0.6519 (2)	0.66612 (12)	0.0230 (4)	
N4	0.37734 (11)	0.8631 (2)	0.52073 (12)	0.0241 (5)	
N5	0.41266 (11)	0.8324 (2)	0.46958 (12)	0.0225 (5)	
N6	0.45817 (12)	0.5646 (2)	0.58278 (13)	0.0264 (5)	
H6AN	0.4389 (19)	0.534 (4)	0.6156 (19)	0.046 (10)*	
H6BN	0.4769 (18)	0.506 (3)	0.5546 (18)	0.044 (10)*	
C1	0.29526 (13)	0.7285 (2)	0.74460 (15)	0.0252 (5)	
C2	0.34215 (12)	0.6435 (2)	0.72408 (14)	0.0220 (5)	
C3	0.35728 (13)	0.5455 (2)	0.77250 (14)	0.0234 (5)	
C4	0.23438 (14)	0.7031 (3)	0.84031 (14)	0.0258 (5)	
C5	0.23232 (13)	0.8209 (3)	0.86942 (15)	0.0269 (6)	
H5	0.265495	0.882624	0.867108	0.032*	
C6	0.18129 (15)	0.8482 (3)	0.90208 (15)	0.0316 (6)	
H6	0.179484	0.929006	0.922122	0.038*	
C7	0.13298 (14)	0.7580 (3)	0.90557 (16)	0.0314 (6)	
H7	0.098412	0.776905	0.928336	0.038*	
C8	0.13510 (15)	0.6400 (3)	0.87575 (17)	0.0352 (7)	
H8	0.101978	0.578122	0.878016	0.042*	
C9	0.18632 (14)	0.6126 (3)	0.84232 (16)	0.0313 (6)	
H9	0.187874	0.532522	0.821286	0.038*	
C10	0.34044 (15)	0.5069 (3)	0.89333 (14)	0.0300 (6)	
H10A	0.306959	0.534045	0.918095	0.045*	0.5
H10B	0.339565	0.415177	0.889350	0.045*	0.5
H10C	0.386706	0.534382	0.920692	0.045*	0.5
H10D	0.381861	0.455024	0.900663	0.045*	0.5
H10E	0.349255	0.573892	0.929408	0.045*	0.5
H10F	0.302114	0.454687	0.898065	0.045*	0.5
C11	0.40348 (15)	0.4365 (3)	0.77530 (15)	0.0290 (6)	
H11A	0.427737	0.446143	0.738586	0.043*	
H11B	0.376096	0.359208	0.766291	0.043*	
H11C	0.437139	0.432114	0.822884	0.043*	
C12	0.35777 (13)	0.7508 (2)	0.62549 (14)	0.0222 (5)	
H12	0.330612	0.818076	0.634577	0.027*	
C13	0.38717 (13)	0.7557 (2)	0.56620 (14)	0.0237 (5)	
C14	0.42913 (13)	0.6708 (2)	0.54780 (13)	0.0212 (5)	
C15	0.44761 (13)	0.7201 (2)	0.48601 (14)	0.0236 (5)	
C16	0.42297 (13)	0.9285 (2)	0.42269 (14)	0.0239 (5)	
C17	0.43325 (16)	0.8949 (3)	0.35769 (15)	0.0341 (6)	
H17	0.433488	0.809000	0.344528	0.041*	
C18	0.44319 (18)	0.9885 (3)	0.31212 (17)	0.0408 (7)	
H18	0.450361	0.966070	0.267463	0.049*	
C19	0.44287 (15)	1.1135 (3)	0.33029 (17)	0.0343 (7)	
H19	0.450448	1.176686	0.298891	0.041*	
C20	0.43135 (14)	1.1456 (3)	0.39494 (15)	0.0283 (6)	
H20	0.429285	1.231722	0.406967	0.034*	
C21	0.42279 (13)	1.0542 (2)	0.44233 (14)	0.0252 (5)	
H21	0.416884	1.076869	0.487512	0.030*	
C22	0.30443 (14)	0.8929 (3)	0.48461 (18)	0.0376 (7)	
H22A	0.279979	0.907024	0.520799	0.056*	
H22B	0.283073	0.822685	0.453602	0.056*	
H22C	0.301822	0.968835	0.455445	0.056*	

### (E)-4-Amino-5-{[(1,5-dimethyl-3-oxo-2-phenyl-2,3-dihydro-1H-pyrazol-4-yl)imino]methyl}-1-methyl-2-phenyl-2,3-dihydro-1H-pyrazol-3-one Atomic displacement parameters (Å^2^)

**Table:**

	*U*^11^	*U*^22^	*U*^33^	*U*^12^	*U*^13^	*U*^23^
O1	0.0366 (11)	0.0269 (10)	0.0438 (12)	0.0139 (8)	0.0232 (9)	0.0129 (9)
O2	0.0314 (10)	0.0231 (9)	0.0313 (10)	0.0013 (7)	0.0166 (8)	−0.0011 (7)
N1	0.0262 (11)	0.0212 (10)	0.0297 (12)	0.0040 (8)	0.0158 (9)	0.0056 (9)
N2	0.0259 (11)	0.0212 (10)	0.0268 (11)	0.0024 (8)	0.0112 (9)	0.0030 (9)
N3	0.0220 (10)	0.0235 (10)	0.0257 (11)	0.0008 (8)	0.0104 (9)	0.0009 (9)
N4	0.0247 (11)	0.0252 (11)	0.0271 (12)	0.0037 (8)	0.0147 (9)	0.0036 (9)
N5	0.0257 (11)	0.0219 (10)	0.0229 (11)	0.0015 (8)	0.0117 (9)	0.0014 (8)
N6	0.0336 (12)	0.0194 (11)	0.0300 (13)	0.0049 (9)	0.0150 (10)	0.0034 (9)
C1	0.0249 (13)	0.0262 (13)	0.0279 (13)	0.0016 (10)	0.0131 (11)	0.0021 (11)
C2	0.0205 (12)	0.0208 (11)	0.0276 (13)	0.0023 (9)	0.0113 (10)	0.0024 (10)
C3	0.0234 (12)	0.0233 (12)	0.0242 (13)	0.0000 (10)	0.0082 (10)	−0.0012 (10)
C4	0.0272 (13)	0.0274 (13)	0.0258 (13)	0.0016 (10)	0.0126 (11)	0.0014 (11)
C5	0.0231 (13)	0.0290 (14)	0.0306 (14)	−0.0025 (10)	0.0108 (11)	−0.0052 (11)
C6	0.0340 (15)	0.0327 (15)	0.0297 (15)	0.0031 (12)	0.0117 (12)	−0.0034 (12)
C7	0.0304 (14)	0.0352 (15)	0.0350 (15)	0.0070 (12)	0.0194 (12)	0.0056 (12)
C8	0.0344 (16)	0.0348 (15)	0.0440 (18)	−0.0013 (12)	0.0237 (14)	0.0041 (13)
C9	0.0316 (14)	0.0259 (13)	0.0420 (17)	0.0008 (11)	0.0192 (13)	0.0010 (12)
C10	0.0331 (15)	0.0333 (15)	0.0265 (14)	0.0036 (11)	0.0132 (12)	0.0065 (11)
C11	0.0334 (14)	0.0288 (14)	0.0264 (14)	0.0073 (11)	0.0110 (11)	0.0005 (11)
C12	0.0230 (12)	0.0204 (12)	0.0260 (13)	0.0014 (9)	0.0113 (10)	0.0006 (10)
C13	0.0231 (12)	0.0222 (12)	0.0273 (13)	0.0022 (10)	0.0098 (10)	0.0028 (10)
C14	0.0211 (12)	0.0209 (12)	0.0228 (12)	−0.0015 (9)	0.0081 (10)	−0.0019 (9)
C15	0.0248 (13)	0.0211 (12)	0.0269 (13)	−0.0019 (10)	0.0107 (10)	−0.0018 (10)
C16	0.0237 (12)	0.0245 (13)	0.0246 (13)	−0.0033 (10)	0.0087 (10)	0.0021 (10)
C17	0.0455 (17)	0.0323 (15)	0.0263 (14)	−0.0004 (13)	0.0129 (13)	−0.0022 (12)
C18	0.0512 (19)	0.0448 (18)	0.0308 (15)	−0.0003 (14)	0.0188 (14)	0.0052 (13)
C19	0.0315 (14)	0.0362 (15)	0.0371 (16)	0.0011 (12)	0.0129 (12)	0.0131 (13)
C20	0.0258 (13)	0.0241 (13)	0.0343 (15)	−0.0009 (10)	0.0075 (11)	0.0060 (11)
C21	0.0217 (12)	0.0269 (13)	0.0260 (13)	0.0013 (10)	0.0054 (11)	0.0016 (10)
C22	0.0264 (14)	0.0427 (17)	0.0475 (19)	0.0063 (12)	0.0169 (13)	0.0162 (14)

### (E)-4-Amino-5-{[(1,5-dimethyl-3-oxo-2-phenyl-2,3-dihydro-1H-pyrazol-4-yl)imino]methyl}-1-methyl-2-phenyl-2,3-dihydro-1H-pyrazol-3-one Geometric parameters (Å, º)

**Table:**

O1—C1	1.232 (3)	C8—H8	0.9500
O2—C15	1.237 (3)	C9—H9	0.9500
N1—N2	1.390 (3)	C10—H10A	0.9800
N1—C1	1.405 (3)	C10—H10B	0.9800
N1—C4	1.425 (3)	C10—H10C	0.9800
N2—C3	1.349 (4)	C10—H10D	0.9800
N2—C10	1.455 (3)	C10—H10E	0.9800
N3—C12	1.297 (3)	C10—H10F	0.9800
N3—C2	1.391 (3)	C11—H11A	0.9800
N4—N5	1.418 (3)	C11—H11B	0.9800
N4—C13	1.424 (3)	C11—H11C	0.9800
N4—C22	1.476 (3)	C12—C13	1.441 (4)
N5—C15	1.379 (3)	C12—H12	0.9500
N5—C16	1.425 (3)	C13—C14	1.357 (4)
N6—C14	1.364 (3)	C14—C15	1.454 (4)
N6—H6AN	0.90 (4)	C16—C17	1.384 (4)
N6—H6BN	0.98 (4)	C16—C21	1.393 (4)
C1—C2	1.447 (4)	C17—C18	1.386 (4)
C2—C3	1.379 (4)	C17—H17	0.9500
C3—C11	1.482 (4)	C18—C19	1.378 (5)
C4—C9	1.378 (4)	C18—H18	0.9500
C4—C5	1.382 (4)	C19—C20	1.384 (4)
C5—C6	1.389 (4)	C19—H19	0.9500
C5—H5	0.9500	C20—C21	1.385 (4)
C6—C7	1.386 (4)	C20—H20	0.9500
C6—H6	0.9500	C21—H21	0.9500
C7—C8	1.390 (4)	C22—H22A	0.9800
C7—H7	0.9500	C22—H22B	0.9800
C8—C9	1.402 (4)	C22—H22C	0.9800
			
N2—N1—C1	109.7 (2)	H10A—C10—H10C	109.5
N2—N1—C4	120.1 (2)	H10B—C10—H10C	109.5
C1—N1—C4	126.0 (2)	H10D—C10—H10E	109.5
C3—N2—N1	108.5 (2)	H10D—C10—H10F	109.5
C3—N2—C10	126.1 (2)	H10E—C10—H10F	109.5
N1—N2—C10	120.3 (2)	C3—C11—H11A	109.5
C12—N3—C2	120.0 (2)	C3—C11—H11B	109.5
N5—N4—C13	103.86 (19)	H11A—C11—H11B	109.5
N5—N4—C22	110.6 (2)	C3—C11—H11C	109.5
C13—N4—C22	114.3 (2)	H11A—C11—H11C	109.5
C15—N5—N4	111.5 (2)	H11B—C11—H11C	109.5
C15—N5—C16	127.7 (2)	N3—C12—C13	118.1 (2)
N4—N5—C16	118.5 (2)	N3—C12—H12	120.9
C14—N6—H6AN	116 (2)	C13—C12—H12	120.9
C14—N6—H6BN	116 (2)	C14—C13—N4	111.0 (2)
H6AN—N6—H6BN	119 (3)	C14—C13—C12	128.2 (2)
O1—C1—N1	123.9 (2)	N4—C13—C12	120.6 (2)
O1—C1—C2	132.3 (3)	C13—C14—N6	129.4 (3)
N1—C1—C2	103.7 (2)	C13—C14—C15	107.8 (2)
C3—C2—N3	122.6 (2)	N6—C14—C15	122.4 (2)
C3—C2—C1	108.7 (2)	O2—C15—N5	126.0 (2)
N3—C2—C1	128.7 (2)	O2—C15—C14	128.4 (2)
N2—C3—C2	109.0 (2)	N5—C15—C14	105.5 (2)
N2—C3—C11	121.7 (2)	C17—C16—C21	120.8 (3)
C2—C3—C11	129.3 (3)	C17—C16—N5	119.0 (2)
C9—C4—C5	121.3 (3)	C21—C16—N5	120.2 (2)
C9—C4—N1	120.3 (2)	C16—C17—C18	118.9 (3)
C5—C4—N1	118.4 (2)	C16—C17—H17	120.5
C4—C5—C6	119.4 (3)	C18—C17—H17	120.5
C4—C5—H5	120.3	C19—C18—C17	121.3 (3)
C6—C5—H5	120.3	C19—C18—H18	119.3
C7—C6—C5	120.3 (3)	C17—C18—H18	119.3
C7—C6—H6	119.8	C18—C19—C20	119.1 (3)
C5—C6—H6	119.8	C18—C19—H19	120.5
C6—C7—C8	120.0 (3)	C20—C19—H19	120.5
C6—C7—H7	120.0	C19—C20—C21	121.0 (3)
C8—C7—H7	120.0	C19—C20—H20	119.5
C7—C8—C9	119.7 (3)	C21—C20—H20	119.5
C7—C8—H8	120.1	C20—C21—C16	118.9 (3)
C9—C8—H8	120.1	C20—C21—H21	120.5
C4—C9—C8	119.3 (3)	C16—C21—H21	120.5
C4—C9—H9	120.4	N4—C22—H22A	109.5
C8—C9—H9	120.4	N4—C22—H22B	109.5
N2—C10—H10A	109.5	H22A—C22—H22B	109.5
N2—C10—H10B	109.5	N4—C22—H22C	109.5
H10A—C10—H10B	109.5	H22A—C22—H22C	109.5
N2—C10—H10C	109.5	H22B—C22—H22C	109.5
			
C1—N1—N2—C3	5.9 (3)	C5—C4—C9—C8	−1.2 (4)
C4—N1—N2—C3	164.2 (2)	N1—C4—C9—C8	178.2 (3)
C1—N1—N2—C10	162.1 (2)	C7—C8—C9—C4	0.7 (5)
C4—N1—N2—C10	−39.7 (3)	C2—N3—C12—C13	179.8 (2)
C13—N4—N5—C15	−5.6 (3)	N5—N4—C13—C14	4.3 (3)
C22—N4—N5—C15	−128.7 (2)	C22—N4—C13—C14	124.9 (3)
C13—N4—N5—C16	−169.6 (2)	N5—N4—C13—C12	−179.4 (2)
C22—N4—N5—C16	67.3 (3)	C22—N4—C13—C12	−58.8 (3)
N2—N1—C1—O1	173.0 (3)	N3—C12—C13—C14	−3.0 (4)
C4—N1—C1—O1	16.3 (4)	N3—C12—C13—N4	−178.6 (2)
N2—N1—C1—C2	−5.3 (3)	N4—C13—C14—N6	170.8 (3)
C4—N1—C1—C2	−162.0 (2)	C12—C13—C14—N6	−5.2 (5)
C12—N3—C2—C3	−174.6 (2)	N4—C13—C14—C15	−1.5 (3)
C12—N3—C2—C1	5.8 (4)	C12—C13—C14—C15	−177.4 (3)
O1—C1—C2—C3	−175.1 (3)	N4—N5—C15—O2	−172.9 (2)
N1—C1—C2—C3	2.9 (3)	C16—N5—C15—O2	−10.8 (4)
O1—C1—C2—N3	4.5 (5)	N4—N5—C15—C14	4.9 (3)
N1—C1—C2—N3	−177.4 (2)	C16—N5—C15—C14	167.0 (2)
N1—N2—C3—C2	−3.9 (3)	C13—C14—C15—O2	175.7 (3)
C10—N2—C3—C2	−158.3 (2)	N6—C14—C15—O2	2.8 (4)
N1—N2—C3—C11	175.5 (2)	C13—C14—C15—N5	−2.0 (3)
C10—N2—C3—C11	21.1 (4)	N6—C14—C15—N5	−174.9 (2)
N3—C2—C3—N2	−179.1 (2)	C15—N5—C16—C17	42.4 (4)
C1—C2—C3—N2	0.5 (3)	N4—N5—C16—C17	−156.5 (2)
N3—C2—C3—C11	1.5 (4)	C15—N5—C16—C21	−137.4 (3)
C1—C2—C3—C11	−178.9 (3)	N4—N5—C16—C21	23.7 (3)
N2—N1—C4—C9	−42.8 (4)	C21—C16—C17—C18	−0.2 (4)
C1—N1—C4—C9	111.6 (3)	N5—C16—C17—C18	179.9 (3)
N2—N1—C4—C5	136.5 (3)	C16—C17—C18—C19	−0.1 (5)
C1—N1—C4—C5	−69.0 (4)	C17—C18—C19—C20	−1.0 (5)
C9—C4—C5—C6	0.7 (4)	C18—C19—C20—C21	2.4 (4)
N1—C4—C5—C6	−178.6 (3)	C19—C20—C21—C16	−2.7 (4)
C4—C5—C6—C7	0.1 (4)	C17—C16—C21—C20	1.6 (4)
C5—C6—C7—C8	−0.5 (4)	N5—C16—C21—C20	−178.5 (2)
C6—C7—C8—C9	0.1 (5)		

### (E)-4-Amino-5-{[(1,5-dimethyl-3-oxo-2-phenyl-2,3-dihydro-1H-pyrazol-4-yl)imino]methyl}-1-methyl-2-phenyl-2,3-dihydro-1H-pyrazol-3-one Hydrogen-bond geometry (Å, º)

*Cg*1 is the centroid of C4–C9 phenyl ring.

**Table:**

*D*—H···*A*	*D*—H	H···*A*	*D*···*A*	*D*—H···*A*
N6—H6*AN*···N3	0.90 (4)	2.30 (4)	2.894 (3)	123 (3)
N6—H6*BN*···O2^i^	0.98 (3)	2.09 (3)	2.953 (3)	147 (3)
C7—H7···O2^ii^	0.95	2.49	3.417 (4)	165
C10—H10*D*···O2^iii^	0.98	2.52	3.472 (4)	164
C12—H12···O1	0.95	2.34	3.022 (3)	128
C17—H17···O2	0.95	2.55	3.013 (4)	110
C11—H11*B*···*Cg*1^iii^	0.98	2.96	3.645 (3)	128

Symmetry codes: (i) −*x*+1, −*y*+1, −*z*+1; (ii) *x*−1/2, −*y*+3/2, *z*+1/2; (iii) *x*, −*y*+1, *z*+1/2.
