# Supplementary material for: Structure of (E)-4-amino-5-{[(1,5-dimethyl-3-oxo-2-phenyl-2,3-di­hydro-1H-pyrazol-4-yl)imino]­meth­yl}-1-methyl-2-phenyl-2,3-di­hydro-1H-pyrazol-3-one: aerial oxidation of 4-amino­anti­pyrine in di­methyl­formamide
Source: Acta Crystallogr E Crystallogr Commun. 2025 Apr 29;81(Pt 5):438–43. doi: 10.1107/S2056989025003676 (PMC12054767; doi:10.1107/S2056989025003676)

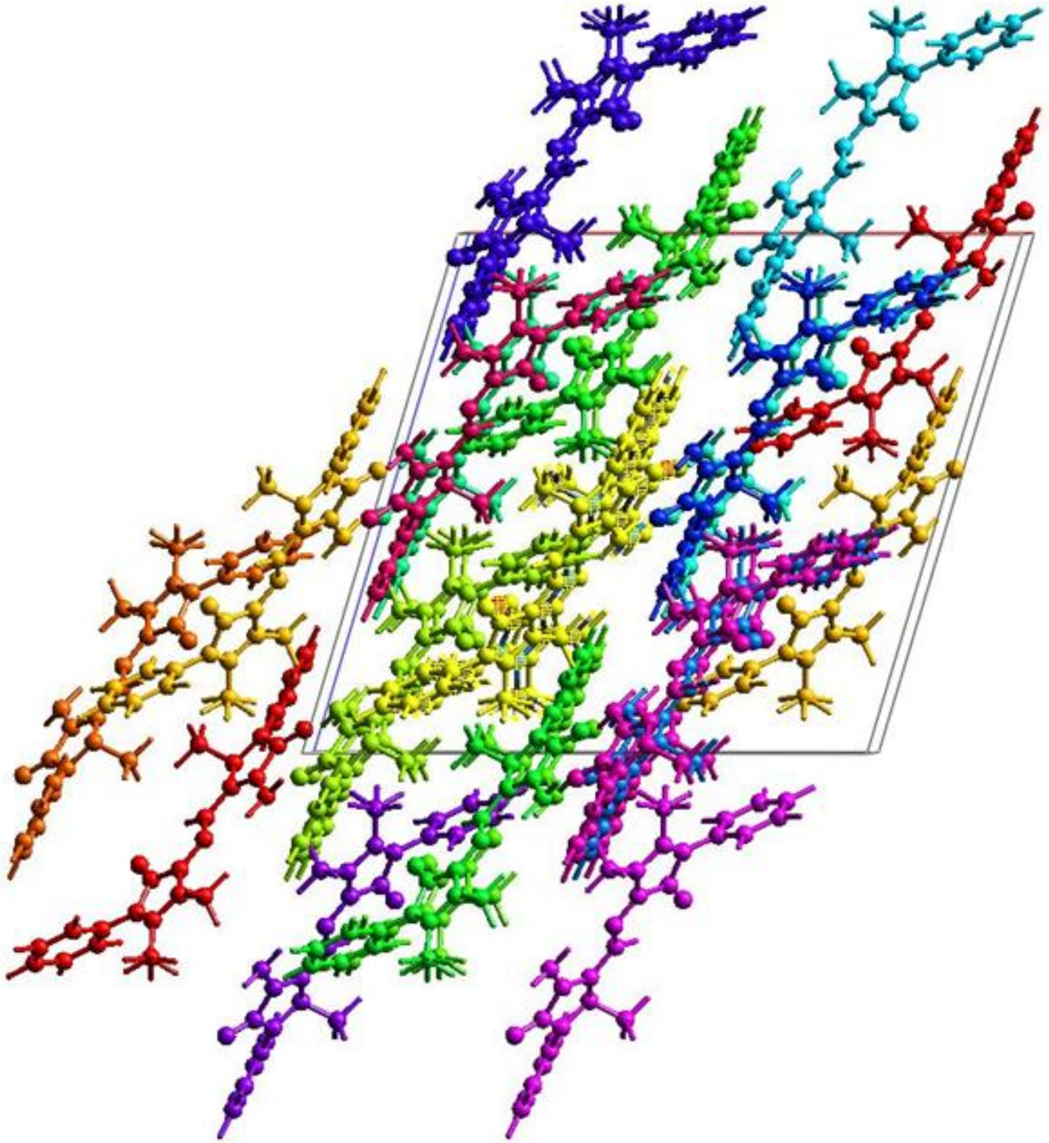

	N	Symop	R	Electron Density	E_ele	E_pol	E_dis	E_rep	E_tot
	1	x+1/2, -y+1/2, z+1/2	15.90	HF/3-21G	0.0	-4.4	0.0	0.0	-2.8
	0	-x, y, -z+1/2	15.74	HF/3-21G	0.0	-0.1	0.0	0.0	-0.1
	1	x+1/2, y+1/2, z	11.44	HF/3-21G	2.0	-0.4	-2.6	0.0	-0.6
	0	x, y, z	10.65	HF/3-21G	0.1	-0.2	-2.2	0.0	-2.1
	0	-x+1/2, y+1/2, -z+1/2	8.41	HF/3-21G	-17.8	-10.9	-66.3	42.4	-50.7
	1	x, -y, z+1/2	11.13	HF/3-21G	-13.9	-4.3	-33.7	43.1	-12.4
	0	x, -y, z+1/2	11.03	HF/3-21G	-2.2	-1.5	-38.7	21.0	-21.2
	1	-x+1/2, -y+1/2, -z	5.73	HF/3-21G	-4.0	-2.6	-52.1	19.8	-36.6
	1	-x, -y, -z	10.86	HF/3-21G	-46.8	-14.9	-14.1	29.4	-46.2
	1	-x, y, -z+1/2	17.99	HF/3-21G	0.0	-0.6	0.0	0.0	-0.4
	1	-x, y, -z+1/2	6.47	HF/3-21G	-5.8	-6.4	-32.5	17.9	-24.8
	1	-x, -y, -z	10.76	HF/3-21G	-10.9	-5.3	-61.1	35.4	-40.9
	0	-x+1/2, y+1/2, -z+1/2	15.50	HF/3-21G	0.0	-0.0	0.0	0.0	-0.0
	0	-x+1/2, -y+1/2, -z	15.51	HF/3-21G	0.0	-1.0	0.0	0.0	-0.6
	0	-x, -y, -z	14.60	HF/3-21G	0.0	-0.2	0.0	0.0	-0.1
	0	-x, y, -z+1/2	12.46	HF/3-21G	0.0	-0.2	0.0	0.0	-0.1
	0	-x+1/2, -y+1/2, -z	12.00	HF/3-21G	0.0	-0.1	0.0	0.0	-0.1

Fig. S1. The colour-coded interaction mappings within a radius of 3.2 Å of a central reference molecule and the various contributions to the total energy (E_{tot}) for compound **I**.

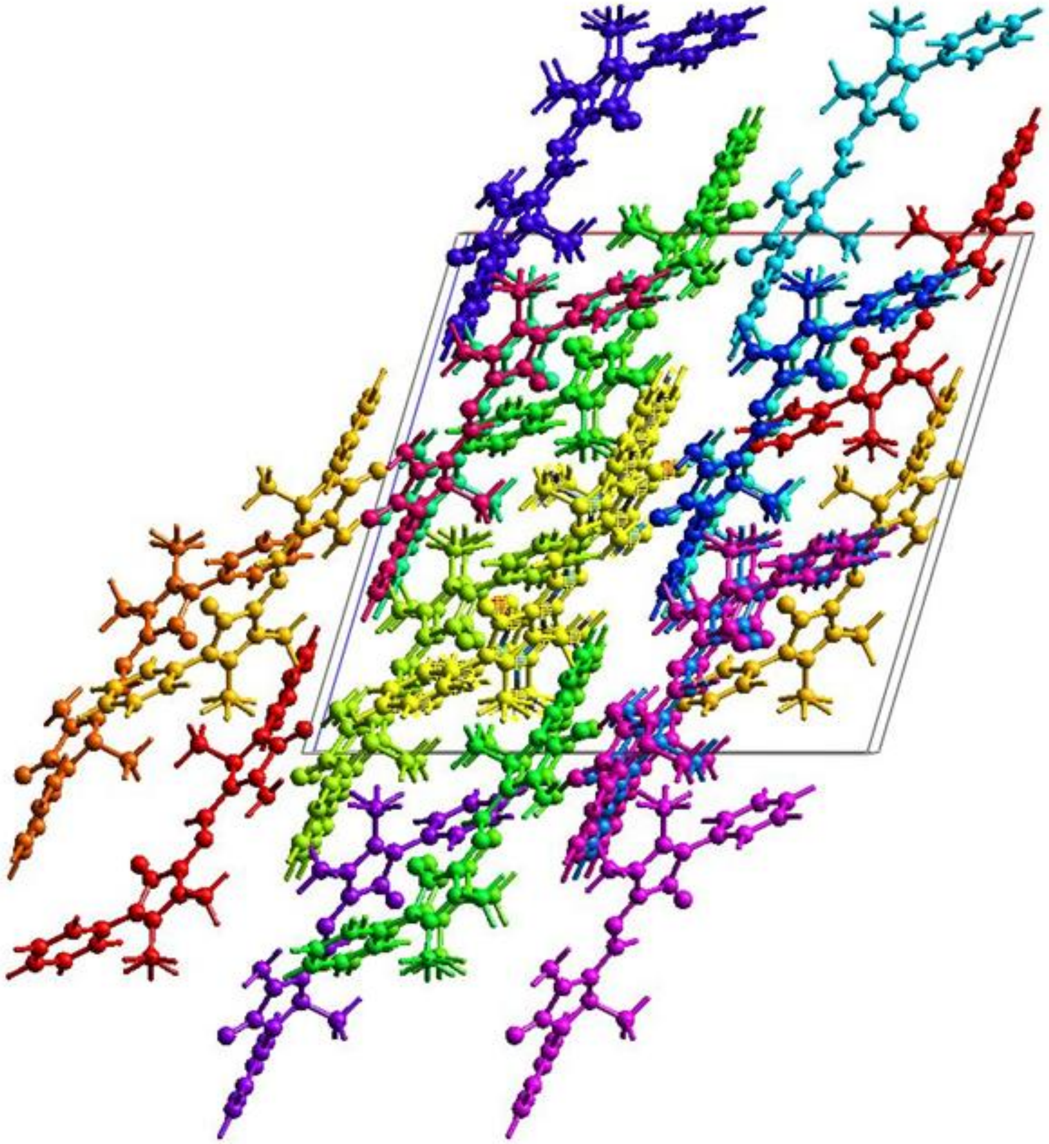

	N	Symop	R	Electron Density	E_ele	E_pol	E_dis	E_rep	E_tot
	1	x+1/2, -y+1/2, z+1/2	15.90	HF/3-21G	0.0	-4.4	0.0	0.0	-2.8
	0	-x, y, -z+1/2	15.74	HF/3-21G	0.0	-0.1	0.0	0.0	-0.1
	1	x+1/2, y+1/2, z	11.44	HF/3-21G	2.0	-0.4	-2.6	0.0	-0.6
	0	x, y, z	10.65	HF/3-21G	0.1	-0.2	-2.2	0.0	-2.1
	0	-x+1/2, y+1/2, -z+1/2	8.41	HF/3-21G	-17.8	-10.9	-66.3	42.4	-50.7
	1	x, -y, z+1/2	11.13	HF/3-21G	-13.9	-4.3	-33.7	43.1	-12.4
	0	x, -y, z+1/2	11.03	HF/3-21G	-2.2	-1.5	-38.7	21.0	-21.2
	1	-x+1/2, -y+1/2, -z	5.73	HF/3-21G	-4.0	-2.6	-52.1	19.8	-36.6
	1	-x, -y, -z	10.86	HF/3-21G	-46.8	-14.9	-14.1	29.4	-46.2
	1	-x, y, -z+1/2	17.99	HF/3-21G	0.0	-0.6	0.0	0.0	-0.4
	1	-x, y, -z+1/2	6.47	HF/3-21G	-5.8	-6.4	-32.5	17.9	-24.8
	1	-x, -y, -z	10.76	HF/3-21G	-10.9	-5.3	-61.1	35.4	-40.9
	0	-x+1/2, y+1/2, -z+1/2	15.50	HF/3-21G	0.0	-0.0	0.0	0.0	-0.0
	0	-x+1/2, -y+1/2, -z	15.51	HF/3-21G	0.0	-1.0	0.0	0.0	-0.6
	0	-x, -y, -z	14.60	HF/3-21G	0.0	-0.2	0.0	0.0	-0.1
	0	-x, y, -z+1/2	12.46	HF/3-21G	0.0	-0.2	0.0	0.0	-0.1
	0	-x+1/2, -y+1/2, -z	12.00	HF/3-21G	0.0	-0.1	0.0	0.0	-0.1

Fig. S1. The colour-coded interaction mappings within a radius of 3.2 Å of a central reference molecule and the various contributions to the total energy (E_{tot}) for compound **I**.

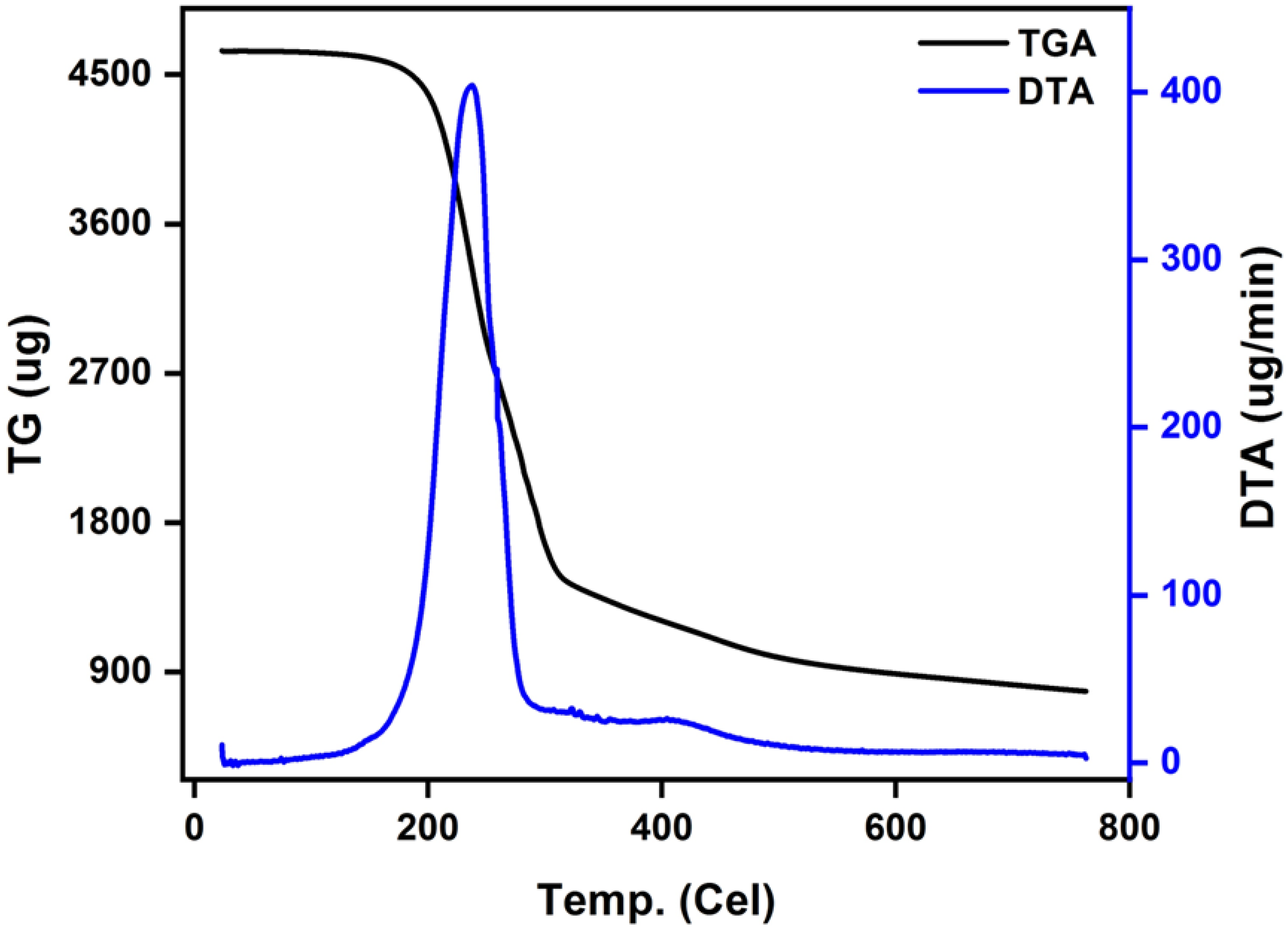

Fig. S2. The thermogravimetric analysis (TGA-black) and the differential thermal analysis (DTA-blue) for compound **I** in the temperature range 25 -- 800 °C.

Search Overview

Search:	search10
Date/Time done:	Mon Mar 17 15:46:47 2025
Database(s):	CSD version 5.46 Updates (Feb 2025)<br>CSD version 5.46 (November 2024)
Restriction Info:	No refcode restrictions applied
Filters:	None
Percentage Completed:	100%
Number of Hits:	13

Single query used. Search found structures that:

match

Query 1

Query 1

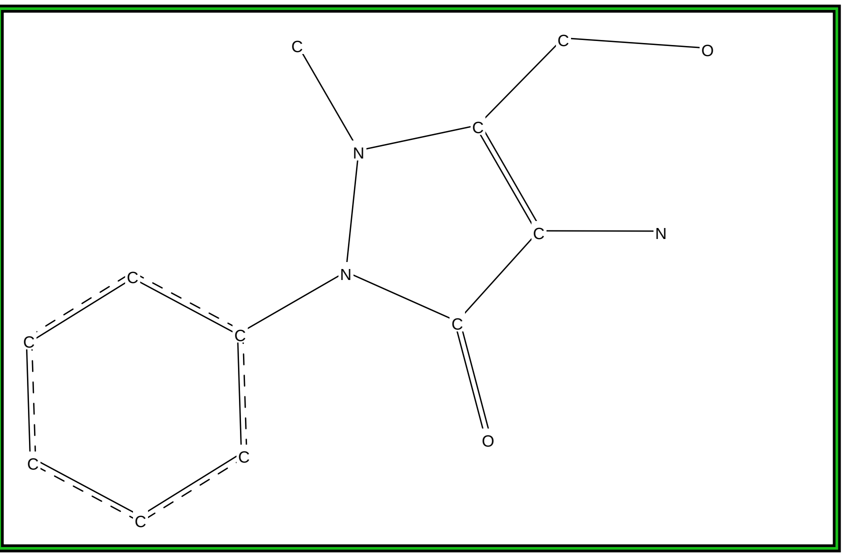

Search: search10 (Mon Mar 17 15:46:47 2025): Hits 1-4

BIYBAM

Reference: B.E.Robertson, S.Hu, K.E.Johnson (1982) *ACA,Ser.2* ,**10,** 19d

Formula: $C_{18} H_{17} N_3 O_2$

Compound Name: 2-Phenyl-1-methyl-3-oxodiazolo(4,5-e)(1',4',7'-4'')methylbenzazepine

Space Group:	P21/c	***Cell:***	***a*** 15.072(4)	***b*** 14.491(4)	***c*** 15.505(3)		
Space Group No.:	14	***(Å,°)***	α 90.00	β 111.15(1)	γ 90.00		
R-Factor (%):	4.60	***Temperature(K):***	295	***Density(g/cm³):***	1.293		

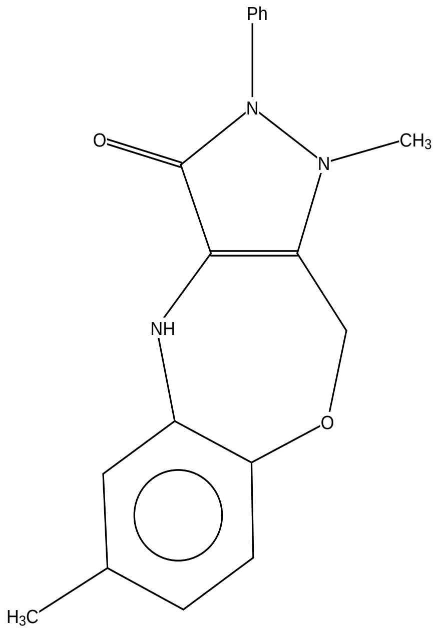

BIYBAM10

Reference: R.J.Barton, K.E.Johnson, B.E.Robertson, F.W.Yerhoff, Shengzhi Hu (1987) *Can.J.Chem.* ,**65,**2082

Formula: $C_{18} H_{17} N_3 O_2$

Compound Name: 1,2,4,10-Tetrahydro-1,6-dimethyl-2-phenyl-3H-pyrazolo(3,4-c)(1,5) benzoxazepin-3-one

Space Group:	P21/c	***Cell:***	***a*** 15.072(4)	***b*** 14.491(4)	***c*** 15.505(3)		
Space Group No.:	14	***(Å,°)***	α 90.00	β 111.15(1)	γ 90.00		
R-Factor (%):	4.60	***Temperature(K):***	295	***Density(g/cm³):***	1.293		

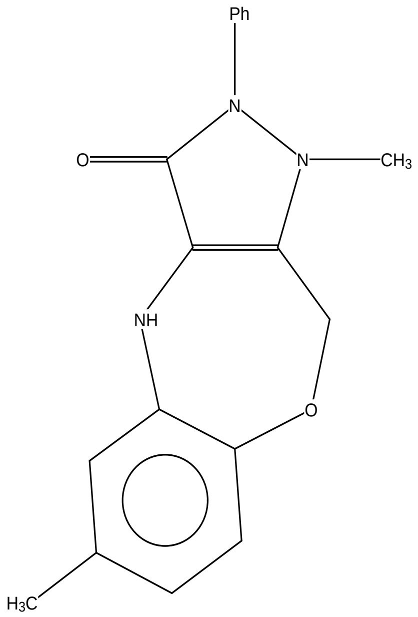

BIYBEQ

Reference: R.J.Barton, B.E.Robertson, S.Hu, K.E.Johnson (1982) *ACA,Ser.2* ,**10,**19

Formula: $C_{19} H_{19} N_3 O_2$

Compound Name: 2-Phenyl-1-methyl-3-oxodiazolo(4,5-e)(1',4',7'-4'')-ethylbenzoxazepine

Space Group:	C2/c	***Cell:***	***a*** 33.768(10)	***b*** 6.678(5)	***c*** 15.836(5)		
Space Group No.:	15	***(Å,°)***	α 90.00	β 115.41(2)	γ 90.00		
R-Factor (%):	4.30	***Temperature(K):***	295	***Density(g/cm³):***	1.324		

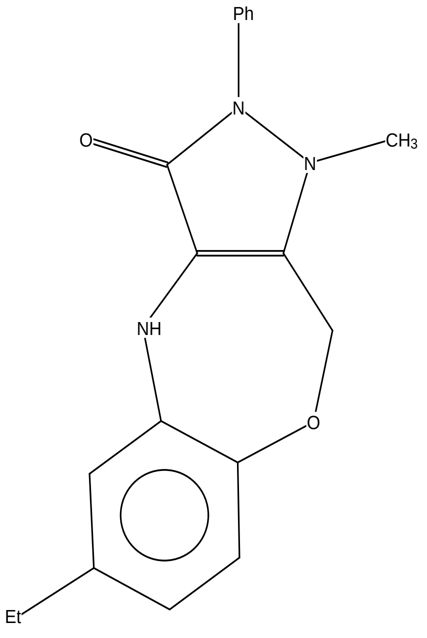

BIYBEQ10

Reference: R.J.Barton, K.E.Johnson, B.E.Robertson, F.W.Yerhoff, Shengzhi Hu (1987) *Can.J.Chem.* ,**65,**2082

Formula: $C_{19} H_{19} N_3 O_2$

Compound Name: 1,2,4,10-Tetrahydro-6-ethyl-1-methyl-2-phenyl-3H-pyrazolo(3,4-c)(1,5) benzoxazepin-3-one

Space Group:	C2/c	***Cell:***	***a*** 33.768(3)	***b*** 6.678(1)	***c*** 15.836(2)		
Space Group No.:	15	***(Å,°)***	α 90.00	β 115.41(1)	γ 90.00		
R-Factor (%):	4.30	***Temperature(K):***	295	***Density(g/cm³):***	1.324		

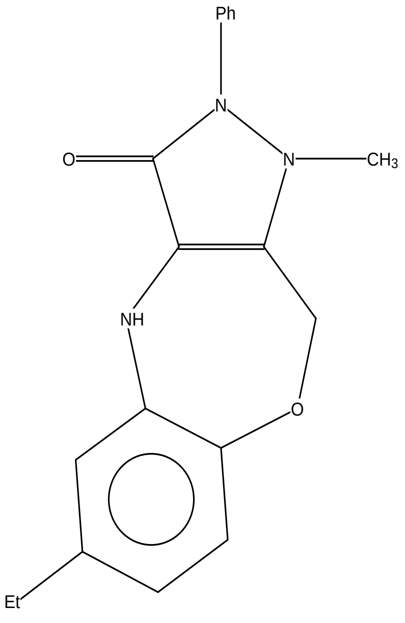

Search: search10 (Mon Mar 17 15:46:47 2025): Hits 5-8

CEXJIB

Reference: S.I.Sampani, E.Loukopoulos, M.Azam, K.Griffiths, A.Abdul-Sada, G.Tizzard, S.Coles, A.Escuer, A.Tsipis, G.E.Kostakis (2018) *CrystEngComm* ,**20,**1411

Formula: $C_{72} H_{64} Cu_4 N_{12} O_{14}$,3($C_2 H_3 N_1$)

Compound Name: bis(μ-5-(oxymethyl)-4-{[(2-oxyphenyl)methylidene]amino}-1-methyl-2-phenyl-1,2-dihydro-3H-pyrazol-3-one)-bis(μ-4-hydroxy-4-oxy-5-{[(2-oxyphenyl)methylidene]amino}-1,5-dimethyl-2-phenylpyrazolidin-3-one)-tetra-copper(ii) acetonitrile solvate

Space Group: C2/c ***Cell:*** ***a*** 21.937(0) ***b*** 18.264(0) ***c*** 18.698(0)
Space Group No.: 15 ***(Å,°)*** α 90.00 β 103.48(0) γ 90.00

R-Factor (%): 2.80 ***Temperature(K):*** 100 ***Density(g/cm³):*** 1.549

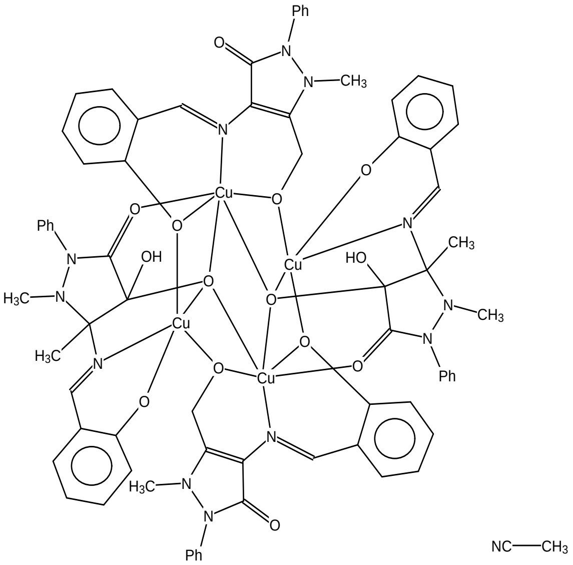

CIHPUF

Reference: Xian-Wen Wang, Yue-Qing Zheng (2007) *Inorg.Chem.Commun.* ,**10,**709

Formula: $C_{36} H_{30} Cu_2 N_6 O_6$

Compound Name: bis(μ_2-(4E)-4-(2-Oxybenzylideneamino)-1,2-dihydro-3-(oxymethyl)-2-methyl-1-phenylpyrazol-5-one-N,O,O,O')-di-copper(ii)

Space Group: C2/c ***Cell:*** ***a*** 27.330(6) ***b*** 7.276(1) ***c*** 19.424(3)
Space Group No.: 15 ***(Å,°)*** α 90.00 β 124.54(1) γ 90.00

R-Factor (%): 5.85 ***Temperature(K):*** 295 ***Density(g/cm³):*** 1.607

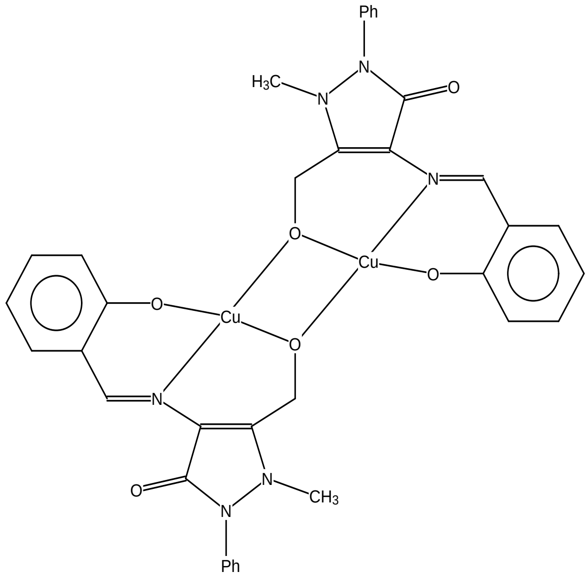

CIHPUF01

Reference: S.Parvarinezhad, M.Salehi, M.Kubicki, Ali Khaleghian (2021) *Appl.Organomet.Chem.* ,**35,**e6443

Formula: $C_{36} H_{30} Cu_2 N_6 O_6$

Compound Name: bis(μ-2-({[1-methyl-5-(oxidomethyl)-3-oxo-2-phenyl-2,3-dihydro-1H-pyrazol-4-yl]imino}methyl)phenolato)-di-copper(ii)

Space Group: I2/a ***Cell:*** ***a*** 19.098(0) ***b*** 7.401(0) ***c*** 22.637(1)
Space Group No.: 15 ***(Å,°)*** α 90.00 β 99.05(0) γ 90.00

R-Factor (%): 5.18 ***Temperature(K):*** 100 ***Density(g/cm³):*** 1.618

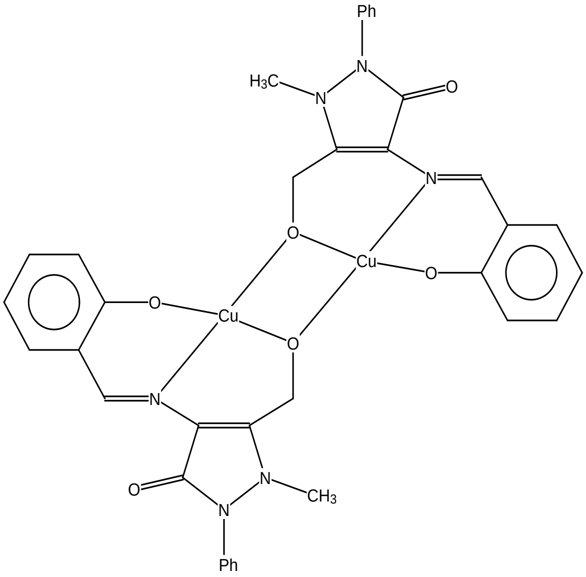

JAXSOU

Reference: S.Parvarinezhad, M.Salehi, M.Kubicki, R.E.malekshah (2022) *J.Mol.Struct.* ,**1260,**132780

Formula: ($C_{19} H_{17} Cu_1 N_3 O_4$)n,0.29($H_2 O_1$)

Compound Name: catena-[(μ-2-methoxy-6-[{[1-methyl-5-(oxidomethyl)-3-oxo-2-phenyl-2,3-dihydro-1H-pyrazol-4-yl]imino}methyl]phenolato)-copper(ii) hydrate]

Space Group: P21/c ***Cell:*** ***a*** 7.847(0) ***b*** 12.586(0) ***c*** 17.084(0)
Space Group No.: 14 ***(Å,°)*** α 90.00 β 97.14(0) γ 90.00

R-Factor (%): 3.59 ***Temperature(K):*** 295 ***Density(g/cm³):*** 1.667

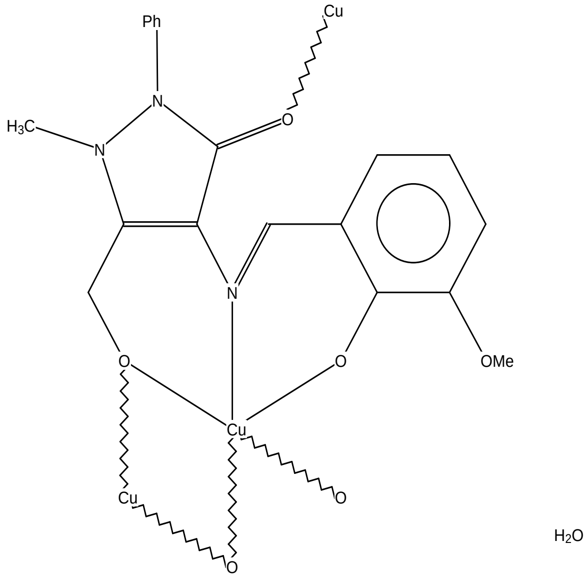

Search: search10 (Mon Mar 17 15:46:47 2025): Hits 9-12

JUNMAI

Reference: E.Loukopoulos, B.Berkoff, K.Griffiths, V.Keeble, V.N.Dokorou, A.C.Tsipis, A.Escuer, G.E.Kostakis (2015) *CrystEngComm* ,**17,**6753

Formula: $C_{76} H_{76} Co_4 N_{12} O_{20}$

Compound Name: tetrakis(μ-2-methoxy-6-(((1-methyl-5-(oxidomethyl)-3-oxo-2-phenyl-2,3-dihydro-1H-pyrazol-4-yl)imino)methyl)phenolato)-tetra-aqua-tetra-cobalt unknown solvate

Space Group:	I41/a	***Cell:***	***a*** 15.063(0)	***b*** 15.063(0)	***c*** 33.631(1)
Space Group No.:	88	***(Å,°)***	α 90.00	β 90.00	γ 90.00

R-Factor (%): 4.85 ***Temperature(K):*** 173 ***Density(g/cm³):*** 1.491

$Co_4[\mu_3\text{-}C_{11}H_6N_3O_3Ph(OMe)Me\text{-}\kappa^1N,\kappa^4O,O']_4[H_2O]_4$

JUNMEM

Reference: E.Loukopoulos, B.Berkoff, K.Griffiths, V.Keeble, V.N.Dokorou, A.C.Tsipis, A.Escuer, G.E.Kostakis (2015) *CrystEngComm* ,**17,**6753

Formula: $C_{76} H_{76} Co_4 N_{12} O_{24}$

Compound Name: tetrakis(μ-2-(((5-(hydroxy(oxido)methyl)-1-methyl-3-oxo-2-phenyl-2,3-dihydro-1H-pyrazol-4-yl)imino)methyl)-6-methoxyphenolato)-tetra-aqua-tetra-cobalt unknown solvate

Space Group:	I41/a	***Cell:***	***a*** 15.014(0)	***b*** 15.014(0)	***c*** 33.654(1)
Space Group No.:	88	***(Å,°)***	α 90.00	β 90.00	γ 90.00

R-Factor (%): 5.15 ***Temperature(K):*** 173 ***Density(g/cm³):*** 1.556

$Co_4[\mu_3\text{-}C_{11}H_6N_3O_4Ph(OMe)Me\text{-}\kappa^1N,\kappa^4O,O']_4[H_2O]_4$

JUNMIQ

Reference: E.Loukopoulos, B.Berkoff, K.Griffiths, V.Keeble, V.N.Dokorou, A.C.Tsipis, A.Escuer, G.E.Kostakis (2015) *CrystEngComm* ,**17,**6753

Formula: $C_{38} H_{33} Co_1 N_6 O_8,2(C_2 H_3 N_1)$

Compound Name: (2-(((1,5-dimethyl-3-oxo-2-phenyl-2,3-dihydro-1H-pyrazol-4-yl)imino) methyl)-6-methoxyphenolato)-(4-(((3-methoxy-2-oxidophenyl) methylidene)amino)-2-methyl-5-oxo-1-phenyl-2,5-dihydro-1H-pyrazole-3-carboxylato)-cobalt(iii) acetonitrile solvate

Space Group:	P-1	***Cell:***	***a*** 12.696(0)	***b*** 14.445(0)	***c*** 22.498(0)
Space Group No.:	2	***(Å,°)***	α 77.80(0)	β 79.84(0)	γ 72.70(0)

R-Factor (%): 7.46 ***Temperature(K):*** 100 ***Density(g/cm³):*** 1.465

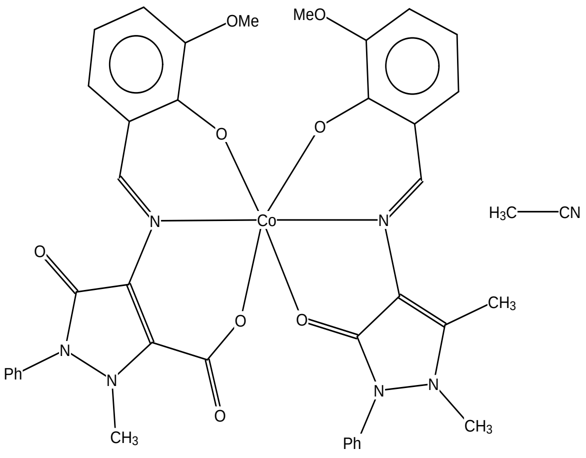

NEQDUM

Reference: Jing Hu, Yun Luo, Min Hou, Jia Jia Qi, Li Li Liang, Wen Ge Li (2022) *Appl.Organomet.Chem.* ,**36,**e6833

Formula: $C_{36} H_{28} Cl_2 Cu_2 N_6 O_6$

Compound Name: bis(μ-4-chloro-2-[{[1-methyl-5-(oxidomethyl)-3-oxo-2-phenyl-2,3-dihydro-1H-pyrazol-4-yl]imino}methyl]phenolato)-di-copper(ii)

Space Group:	P21/n	***Cell:***	***a*** 7.054(0)	***b*** 8.367(0)	***c*** 28.334(1)
Space Group No.:	14	***(Å,°)***	α 90.00	β 95.36(0)	γ 90.00

R-Factor (%): 5.20 ***Temperature(K):*** 172 ***Density(g/cm³):*** 1.673

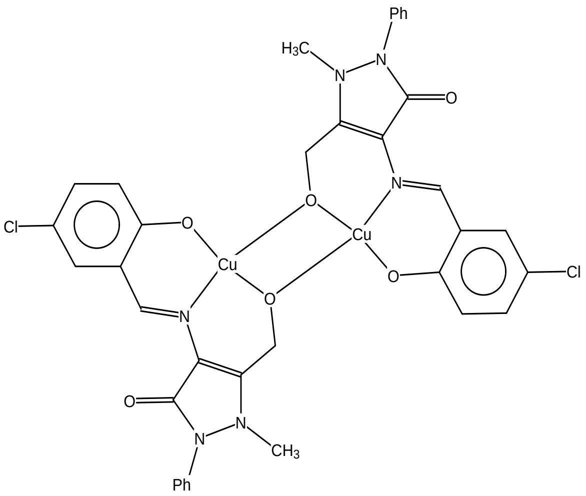

Search: search10 (Mon Mar 17 15:46:47 2025): Hit 13

VIBYEN

Reference:	S.Layek, Rakesh Ganguly, D.D.Pathak (2018) *J.Organomet.Chem.* ,**870,**16
Formula:	$(C_{19} H_{17} Cu_1 N_3 O_4)n$
Compound Name:	catena-[(μ-2-methoxy-6-[{[1-methyl-5-(oxidomethyl)-3-oxo-2-phenyl-2,3-dihydro-1H-pyrazol-4-yl]imino}methyl]phenolato)-copper(ii)]

Space Group:	P21/c	***Cell:***	***a***	7.855(0)	***b***	12.628(0)	***c***	17.103(0)
Space Group No.:	14	***(Å,°)***	α	90.00	β	97.09(0)	γ	90.00

R-Factor (%):	6.90	***Temperature(K):***	153	***Density(g/cm³):***	1.637

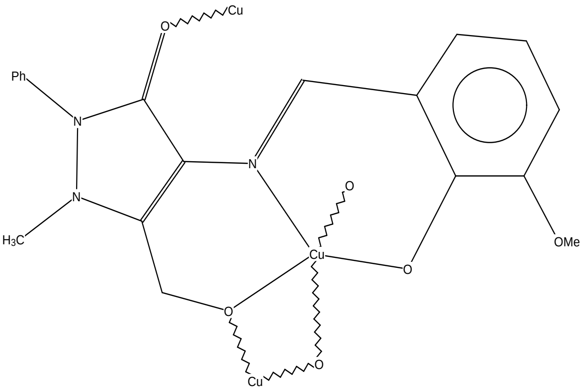

Supplement: Supplementary file 3 [file e-81-00438-sup3.pdf]
